# Expression of Leukemia-Associated Nup98 Fusion Proteins Generates an Aberrant Nuclear Envelope Phenotype

**DOI:** 10.1371/journal.pone.0152321

**Published:** 2016-03-31

**Authors:** Birthe Fahrenkrog, Valérie Martinelli, Nadine Nilles, Gernot Fruhmann, Guillaume Chatel, Sabine Juge, Ursula Sauder, Danika Di Giacomo, Cristina Mecucci, Jürg Schwaller

**Affiliations:** 1 Institute of Molecular Biology and Medicine, Université Libre de Bruxelles, Charleroi, Belgium; 2 Department of Biomedicine, University Children’s Hospital Basel, Basel, Switzerland; 3 Biozentrum, Microscopy Center, University of Basel, Basel, Switzerland; 4 Hematology and Bone Marrow Transplantation Unit, University of Perugia, Perugia, Italy; Brunel University, UNITED KINGDOM

## Abstract

Chromosomal translocations involving the nucleoporin *NUP98* have been described in several hematopoietic malignancies, in particular acute myeloid leukemia (AML). In the resulting chimeric proteins, Nup98's N-terminal region is fused to the C-terminal region of about 30 different partners, including homeodomain (HD) transcription factors. While transcriptional targets of distinct Nup98 chimeras related to immortalization are relatively well described, little is known about other potential cellular effects of these fusion proteins. By comparing the sub-nuclear localization of a large number of Nup98 fusions with HD and non-HD partners throughout the cell cycle we found that while all Nup98 chimeras were nuclear during interphase, only Nup98-HD fusion proteins exhibited a characteristic speckled appearance. During mitosis, only Nup98-HD fusions were concentrated on chromosomes. Despite the difference in localization, all tested Nup98 chimera provoked morphological alterations in the nuclear envelope (NE), in particular affecting the nuclear lamina and the lamina-associated polypeptide 2α (LAP2α). Importantly, such aberrations were not only observed in transiently transfected HeLa cells but also in mouse bone marrow cells immortalized by Nup98 fusions and in cells derived from leukemia patients harboring Nup98 fusions. Our findings unravel Nup98 fusion-associated NE alterations that may contribute to leukemogenesis.

## Introduction

Chromosomal translocations of the nucleoporin *NUP98* have been described in several hematopoietic malignancies, in particular *de novo* and therapy-related acute myeloid leukemia (AML) [1, 2]. These chromosomal translocations generate Nup98 chimera, in which the N-terminal region of *NUP98* is fused to the C-terminal region of about 30 different fusion partners, including several homeodomain (HD) transcription factors [1, 2]. Nup98 is a component of the nuclear pore complex (NPC), which mediates trafficking between the nucleus and the cytoplasm of interphase cells [3, 4]. It has been characterized as a mobile nucleoporin, which dynamically associates with NPCs in a transcription-dependent manner [5, 6]. Within the NPC, Nup98 is anchored to its center [7, 8], where it contributes to both protein import [9] as well as mRNA export [10–12] and the NPC's permeability barrier [13]. Nup98 also influences gene expression: it is recruited to promoters of developmentally regulated genes in *Drosophila* [14, 15] and in human embryonic stem cells [16], it promotes epigenetic transcriptional memory for interferon-γ-inducible genes [17] and prevents *p21*^*WAF1*^ mRNA degradation by the exosome [18]. During mitosis, Nup98 regulates mitotic spindle assembly [19] and the timing of mitotic exit via the anaphase-promoting complex [20, 21].

Nup98 is comprised of two major domains: a N-terminal GLFG (glycine-lysine-phenylalanine-glycine) repeat domain, which mediates the binding of Nup98 to soluble nuclear transport receptors, and a C-terminal auto-proteolytic domain [22]. All leukemia-associated *NUP98* fusions preserve the GLFG repeats of Nup98, which can act as both transcriptional co-activators through recruitment of p300/CBP [23] and co-repressors through recruitment of HDAC1 [24]. The FG domain of *NUP98* is fused in frame to partner genes that include ten HD genes and over 20 non-HD genes. The non-HD fusion partners often contain histone "reading" and "writing" domains, such as PHD fingers and SET domains [1, 2]. The best-studied chimera is *NUP98-HOXA9* resulting from t(7;11) mostly associated with AML and chronic myeloid leukemia (CML) in blast crisis [23, 25–31].

Transcriptional targets of several Nup98 fusion proteins related to immortalization are relatively well studied, however, whether the fusions might have other cellular effects remains unclear. To expand our knowledge in this respect, we have compared sub-cellular localization and the behavior during interphase and mitosis of a series of Nup98 fusion proteins with HD and non-HD partners. We found that transforming Nup98 chimeras consistently provoked an aberrant nuclear envelope (NE) phenotype with morphological alterations in the nuclear lamina and the lamina-associated polypeptide 2α (LAP2α) suggesting that these Nup98 fusion-associated NE alterations may contribute to leukemogenesis.

## Material and Methods

All experimental procedures were carried out at room temperature unless otherwise stated.

### Cell culture and transfections

HeLa cells were grown in Dulbecco’s modified Eagle’s medium (DMEM) supplemented with 10% fetal bovine serum (FBS) plus 100 U/ml penicillin and 100 U/ml streptomycin. Cells were transfected using Turbofect (Fermentas, Thermo Fisher Scientific, Gent, Belgium) following the manufacturer’s instructions.

### Constructs

For all constructs, inserts were amplified by PCR or subcloned by enzymatic digestion. All constructs were verified by DNA sequencing. N-terminally tagged GFP-Nup98, GFP-Nup98-HOXA9, GFP-Nup98-HHEX, GFP-Nup98-LEDGF and GFP-HOXA9 were cloned into *XhoI/BamHI* cut pEGFP-C1 (Clontech, Palo Alto, CA, USA), GFP-Nup98-PMX1 was cloned *XbaI/XhoI*, GFP-Nup98-HOXA10 *PstI/XmaI*, GFP-Nup98-NSD1 and GFP-Nup98-NSD3 were inserted into *HindIII/BamHI* cut pEGFP-C1. GFP-Nup98-JARID1A, GFP-Nup98-PHF23, and GFP-Nup98-RARG were cloned into *EcoRI/XhoI* cut pEGFP-C1. The respective cDNAs were provided by Keith Humphries (Terry Fox Laboratories, Vancouver, Canada), Jan de Rjick (KU Leuven, Belgium) and Shuo Dong (Baylor College of Medicine, Houston, TX, USA) or generated by breakpoint cloning from leukemia patients ([32]; J.S. & C.M. unpublished).

GFP-Nup98-HHEX ΔHD was generated by *XbaI*-mediated excision, GFP-Nup98-HOXA9 ΔFG and Nup98-HHEX ΔFG by excision using *SpeI*. GFP-Nup98-HOXA9 N51S, GFP-Nup98-PMX1 N51S and GFP-Nup98-JARID1A W1625A were generated by site-directed mutagenesis using *Pfu* Ultra DNA polymerase (Stratagene, Agilent Technologies, Diegem, Belgium) following the manufacturer’s instructions. Primers used were: Nup98-HOXA9 forward 5'-GATCTGGTTCCAGAGCCGCAGGATGAAAA-3'; reverse 5'-TTTTCATCCTGCGGCTCTGGAACCAGATC-3'; Nup98-PMX1 forward 5'-TGGTTTCAGAGCCGAAGAGCCA-3'; reverse 5'-TGGCTCTTCGGCTCTGAAACCA-3'; Nup98-JARID1A forward 5'-GGACAAGGTAGACGCGGTACAATGTGATGG-3'; reverse 5'-CCATCACATTGTACCGCGTCTACCTTGTCC-3'. All constructs used in this study are listed in S1 Table.

### Antibodies

The following primary antibodies were used for immunofluorescence microscopy: human anti-centromere (CREST serum 1:3, Antibodies Inc., 15-234-001; Antibodies Inc., Davis, CA, USA), mouse monoclonal anti-lamin A/C (1:30; Abcam, ab40567; Abcam, Cambridge, UK), monoclonal anti-LAP2α (1:10; clone 15/2, kind gift of Dr. Roland Foisner, Max F. Perutz Laboratories, Vienna, Austria), monoclonal antibody mAb414 (1:2000; Covance, MMS-120R; Covance, Emeryville, CA, USA), monoclonal anti-Hec1 (1:200; clone 3G9, Abcam, ab3613), and rabbit polyclonal anti-LAP2α (1:1000; Abcam, ab5162), polyclonal anti-lamin A (1:500; Sigma-Aldrich, L1293; Sigma-Aldrich, Diegem, Belgium), polyclonal lamin B1 (Abcam, ab16048), polyclonal anti-Sun1 (1:1000, kind gift of Dr. Ulrike Kutay, ETH Zurich, Switzerland), polyclonal anti-Sun2 (1:100; Sigma-Aldrich, HPA001209), polyclonal anti-emerin (1:1000; Bethyl Laboratories, A304-491A; ImTec Diagnostics, Antwerpen, Belgium), as well as polyclonal anti-Nesprin-2 (1:50, kind gift of Dr. Iakowos Karakesisoglou, Durham University, UK). Secondary antibodies were the corresponding goat anti-mouse IgG Alexa 568 (1:1000; Invitrogen), goat anti-mouse IgG Alexa 555 (1:1000; Invitrogen), goat anti-rabbit IgG Alexa 568 (1:1000; Invitrogen), goat anti-mouse IgG Alexa 633 (1:350; Invitrogen), and goat anti-rabbit IgG Alexa 633 (1:350; Invitrogen).

### Generation of stable cell lines

pcDNA4-GFP-Nup98 and pcDNA4-GFP-Nup98-HOXA9 were generated by *BamHI*/*EcoRI* digestion of pEGFP-Nup98 and pEGFP-Nup98-HOXA9, respectively. The GFP-Nup98 and GFP-Nup98-HOXA9 fragments included a stop codon at the end of the sequence and were inserted into *BamHI/EcoRI* cut pcDNA4-TO-myc-His B (Invitrogen). HeLa T-Rex cells (Invitrogen) were grown in minimal essential medium (MEM) containing GlutaMAX^™^ (Invitrogen) supplemented with 10% FBS (Biochrom), 100 U/ml penicillin and 100 U/ml streptomycin (Invitrogen) and transfected with pcDNA4-GFP-Nup98 or pcDNA4-GFP-Nup98-HOXA9 using Turbofect. Cells were incubated for 48 h and diluted 1:10 into fresh MEM. After another 24 h, the medium was exchanged by fresh medium containing 5 μg/ml blasticidin (Invitrogen) and 200 μg/ml zeocin (Invivogen, Toulouse, France). After 2 to 3 weeks, single colonies were selected and tested for expression of GFP-Nup98 or GFP-Nup98-HOXA9 by addition of 1 μg/ml tetracycline (Sigma–Aldrich) for 24 h and subsequent analysis by Western blotting. Selected clones were maintained stable in MEM containing 5 μg/ml blasticidin and 200 μg/ml zeocin.

### Immunofluorescence microscopy of HeLa cells

HeLa cells were grown on glass coverslips and fixed in 2% formaldehyde for 15 min, washed three times for 10 min with PBS, and permeabilized with PBS containing 1% bovine serum albumin (BSA) and 0.2% Triton X-100 for 10 min on ice. Next the cells were washed three times for 10 min in PBS containing 1% BSA, incubated with the appropriate primary antibodies for 1 h, washed three times in PBS containing 1% BSA, incubated with the appropriate secondary antibodies for 1 h, washed four times for 10 min with PBS, mounted with a drop of Mowiol-4088 (Sigma-Aldrich) containing DAPI (1μg/ml), and stored at 4°C until viewed. Cells were imaged using either a Leica TCS NT/SP5 (Leica, Vienna, Austria) or a Zeiss LSM-710 (Zeiss, Oberkochen, Germany) confocal laser-scanning microscope. Images were recorded using the microscope system software and processed using ImageJ (http://imagej.nih.gov) and Adobe Photoshop (Adobe Systems, Mountain View, CA, USA).

### Time-lapse imaging

For live cell imaging, cells were grown on CELLview^™^ cell culture dishes with glass bottom (Greiner Bio-One, Vilvoorde, Belgium). Cells were transfected with 200 ng of plasmid DNA encoding GFP, GFP-NUP98, GFP-NUP98-HOXA9, and GFP-NUP98-PMX1, respectively, using Turbofect transfection reagent (Thermo Fisher Scientific). Six hours after transfection, cells were synchronized at the G1/S transition by two sequential 16 h blocks with 2 mM thymidine separated by an 8 h release in between. The release after the second thymidine block was performed in DMEM medium. Approximately 2 h before acquiring images, medium was removed and replaced by CO_2_-independent medium without phenol red. Cells were placed into a 37°C pre-heated incubation chamber of an inverted Zeiss Observer Z1 microscope. GFP and differential interference contrast (DIC) images were acquired every 15 min during 20 h using a 20x objective with an AxioCam HRm camera. Images were recorded using Axiovision 4.8.2 software (Zeiss) and analyzed by ImageJ.

### Flow cytometry

HeLa cells were transfected with pEGFP, pEGFP-Nup98 and pEGFP-Nup98-HOXA9 in equimolar range using Turbofect transfection reagent. Cells were synchronized by a double thymidine block 6 h after transfection, using a final concentration of 2 mM thymidine. After 16 h the medium containing thymidine was removed, the cells were washed twice with PBS and kept in complete DMEM medium. After 8 h, thymidine was added to a final concentration of 2 mM for a second 16 h interval. The cells were next washed twice with PBS, kept in complete DMEM medium and were harvested by trypsinization 0, 9 and 13 h after release from thymidine. Cells were fixed in 70% ethanol (at -20°C) and kept overnight. The fixed cells were centrifuged, washed with PBS and allowed to rehydrate for 15 min before removal of PBS. Next, the cells were incubated in RNAse A solution (Invitrogen) at 0.2 mg/ml for 5 min and incubated for 15 min with propidium iodide (PI) staining solution (50 mg/ml PI, 0.1% Triton X-100) at 37°C in the dark. Cells were stored at 4°C in the dark until flow cytometric acquisition on a FACS Canto II machine (BD Biosciences, Erembodegem, Belgium). Data were processed using FlowJo V7.6.1 software (Tree Star, Inc., Ashland, OR, USA).

### Production of retrovirus and transduction of bone marrow cells

The retroviral expression vectors (pMSCV-GFP-NUP98/HOXA9-neo, pMSCV-NUP98/HOXA9-neo and pMSCV-NUP98/HHEX-neo) have been previously described [32]. Retroviral particles were produced by transient transfection of the respective viral vector with the ecotropic packaging vector pIPAK6 in a ratio of 2:1 in HEK-293T cells (kept in DMEM, 10% serum) as previously described [32, 33]. 48 h and 72 h after transduction the virus was harvested and filtered through a 45 μm PVDF-filter and immediately used. Lineage marker-negative bone marrow stem and progenitor cells from healthy 6–10 week old C57/B6 mice were enriched using the Cell Mag Kit (R&D systems, Minneapolis, MN, USA) following the manufacturers protocol. Lin- cells were incubated for 24 h in medium containing IL3, IL6 and mSCF before spinoculation at 2.500 rpm at 30°C for 90 min, that was repeated once 24 h after the first round.

### Culture of mouse and human hematopoietic cell lines

Transduced mouse bone marrow stem and progenitor cells were expanded by serial plating in growth-factor containing semisolid medium (MethoCult GF M3534, Stem Cell Technologies, Vancouver, Canada) for four rounds at 5% CO_2_ at 37°C. Next the cells were harvested and maintained in liquid cultures in medium containing IL3, IL6 and mSCF as described previously [32].

### Immunostaining of bone marrow cells

The cells were harvested by centrifugation and washed twice with ice-cold PBS. 150x 10^3^ cells were resuspended in 100 μl PBS and spun onto microscopy-slides (Shandon Cytoslides, coated, Thermo Fisher Scientific) for 3 min at 300 rpm using a Shandon CytoSpin3 apparatus. Slides were dried at room temperature for two days and stored at -80°C. Before staining, cells were fixed in 2% paraformaldehyde for 15 min, permeabilized in TBST with 0.5% Triton X-100 for 10 min and blocked with TBST plus 5% BSA (blocking buffer) for 1 h and finally incubated with anti-LAP2α (clone 15/2, kindly provided by Roland Foisner, Max F. Perutz Laboratories, Vienna, Austria) 1:10 diluted in blocking buffer overnight at 4°C. The cells were then washed three times for 5 min with blocking buffer before application of the secondary antibody (AlexaFluor-555, Invitrogen) diluted 1:1.000 in blocking buffer for 1 h at room temperature. Double labeling were done sequentially and after washing three times 5 min with blocking buffer the anti-laminA/C (Santa Cruz sc-6215, diluted 1:100 in blocking buffer; Santa Cruz Biotechnology, Heidelberg, Germany) or anti-laminB1 antibodies (Abcam ab16048, diluted 1:500 in blocking buffer) were applied to the cells over night at 4°C. Secondary antibodies (AlexaFluor-647 for sc-6215 or AlexaFluor-555 for ab16048, diluted 1:1.000 in blocking buffer) were applied for 1 h at room temperature. To counterstain the nuclei, cells were incubated in DAPI (1 μg/ml) in PBS for 5 min. Cells were then washed in five times 5min with PBS, mounted with CALBIOCHEM mounting medium (EMD Millipore, Schaffhausen, Switzerland) and finally sealed with nail polish. Stained cells were investigated using a Nikon Ti-Eclipse microscope (Nikon AG, Egg, Switzerland) with filters for DAPI, FITC and TRITC at 100x magnification as well as a Zeiss LSM710 confocal microscope at 63x magnification. Image post-processing was done in Olympus CellSens (Olympus Schweiz AG, Volketswil, Switzerland), Imaris (Bitplane AG, Zurich, Switzerland) and Image J software.

### Electron microscopy

HeLa cells were transiently transfected with pEGFP-Nup98-HOXA9 and pEGFP-Nup98-HHEX, respectively, for 24 hours and HeLa T-Rex cells were treated with 1 μg/ml tetracycline for 24 h to express GFP-Nup98-HOXA9. Cells were harvested using a cell scraper, pelleted and washed once in PBS. Mouse bone marrow cells were harvested by centrifugation 48 h after viral transduction. Cells were fixed in Karnofski solution (3% paraformaldehyde, 0.5% glutaraldehyde in 10 mM PBS, pH 7.4) for 1 h, washed once in PBS and post-fixed first in 1% reduced osmium tetroxide (containing 1.5% potassium ferricyanide) for 40 min and subsequently in 1% osmium tetroxide for another 40 min. After washing in water, fixed samples were dehydrated, embedded in Epon resin, and processed for EM as described [34]. EM micrographs were recorded on a Phillips CM-100 or on a FEI Morgagni 268D transmission electron microscope (FEI/Philipps Europe, Eindhoven, Netherlands) equipped with a CCD camera at an acceleration voltage of 80 kV. Images were recorded using the system software and processed using Adobe Photoshop.

### Reverse-transcription quantitative PCR

RNA was extracted using TRIZOL (Life Technologies; Thermo Fisher Scientific, Gent, Belgium) according to the manufacturer’s protocol. Subsequent cDNA synthesis was done with a reverse transcriptase kit (Applied Biosystems; Thermo Fisher Scientific, Gent, Belgium) and mRNA expression was measured in triplicates by qRT-PCR using SYBR-Green on an ABI prism 7700 Sequence Detection System (Applied Biosystems). All results were calibrated with GAPDH and calculated as DDCT values. The sequences of the respective primers are provided in S2 Table.

### Patient samples

Patients were referred to the Laboratory of Cytogenetics and Molecular Genetics of the Hematology Department at the University of Perugia. Hematological and cytogenetic features of the patient samples are listed in S3 Table. All patients gave their written informed consent to sample collection and biological analyses in accordance with the Declaration of Helsinki. The study was approved by the Bioethics Committee of the University of Perugia (Prot.1.X.2011).

## Results

### Nup98 fusion proteins localize to the nucleus in different patterns

**Fig 1:**
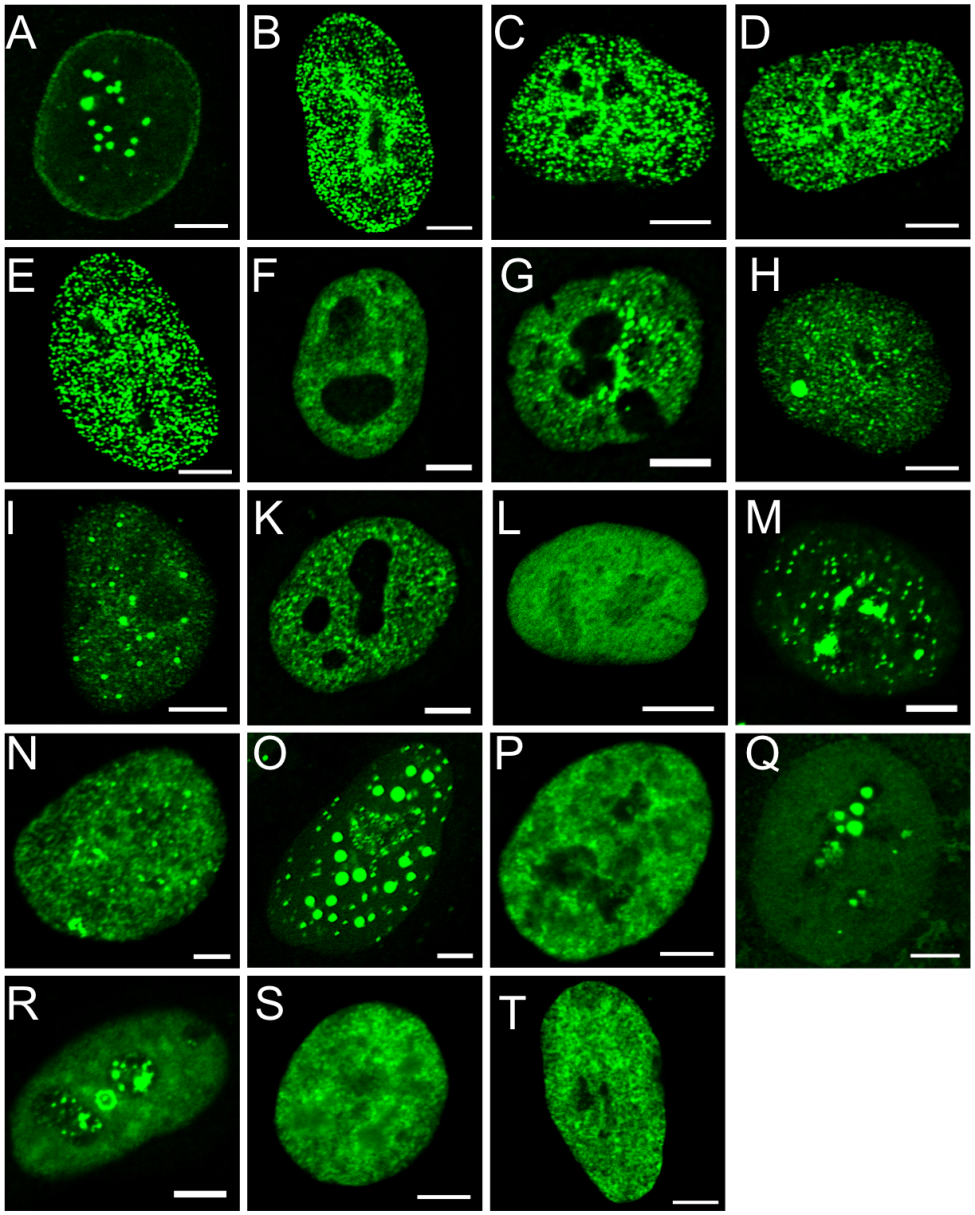
Localization of Nup98 fusion proteins. HeLa cells were transiently transfected with GFP constructs and visualized after 24 hours by direct fluorescence microscopy. All fusion proteins localize to the nucleus. (**A**) GFP-Nup98 is found at the nuclear rim and in the nucleoplasm, whereas Nup98 homeodomain fusions exhibit a punctate pattern: (**B**) GFP-Nup98-HOXA9, (**C**) GFP-Nup98-HOXA10, (**D**) GFP-Nup98-HHEX, and (**E**) GFP-Nup98-PMX1. Nup98 fusions with other chromatin-binding motifs show a different punctate distribution: (**F**) GFP-Nup98-JARID1A, and (**G**) GFP-Nup98-PHF23 (**H**) GFP-Nup98-NSD1, (**I**) GFP-Nup98-NSD3 and (**K**) GFP-Nup98-RARG. Nup98 fused to partners that lack chromatin-binding domains localize more dispersed to the nucleoplasm: (**L**) GFP-Nup98-LEDGF. Disruption of the FG, the HD or the PHD domain disrupts the localization of the Nup98 chimeras: (**M**) GFP-Nup98-PMX1 N51S, (**N**) GFP-Nup98-HOXA9 ΔFG, (**O**) GFP-Nup98-HOXA9 N51S, (**P**) GFP-Nup98-HHEX ΔFG, (**Q**) GFP-Nup98-HHEX ΔHD, and (**R**) GFP-Nup98-JARID1A W1625A. (**S**) GFP-HOXA9 and (**T**) GFP-HHEX localize to the nucleoplasm. Shown are representative confocal images. Scale bars, 5 μm.

Nup98 is known to localize to NPCs and the nucleoplasm [5, 7, 35], while Nup98-HD fusion proteins were previously found in a distinctive punctate pattern throughout the nuclear interior with exclusion from the nucleolus [23, 32, 36]. We first asked whether Nup98 chimeras in which Nup98 is fused to non-HD partners might adapt a similar speckled pattern in the nucleus as the Nup98-HD chimeras. To determine the localization, GFP-tagged fusion proteins (Table 1) were visualized in transiently transfected HeLa cells. GFP-Nup98 (Fig 1A) localized to the nuclear rim in a pattern typical for nucleoporins, to the nucleoplasm, and to nuclear foci, also referred as “GLFG bodies”, as previously described [5, 23, 36]. The fusion proteins GFP-Nup98-HOXA9 (Fig 1B), GFP-Nup98-HOXA10 (Fig 1C), GFP-Nup98-HHEX (Fig 1D) and GFP-Nup98-PMX1 (Fig 1E) were present in a characteristic speckled pattern in the nucleus, as previously seen for GFP-Nup98-HOXA9, GFP-Nup98-HHEX, and GFP-Nup98-PMX1 [23, 32, 36]. In contrast to the Nup98-HD fusion proteins, Nup98 chimeras with PHD finger, zinc-finger or SET domain proteins, such as GFP-Nup98-JARID1A (Fig 1F), GFP-Nup98-PHF23 (Fig 1G), GFP-Nup98-NSD1 (Fig 1H), or GFP-Nup98-NSD3 (Fig 1I), exhibited a nuclear localization with a different, finer punctate pattern very similar as recently described for the GFP-Nup98-RARG fusion (Fig 1K) [37]. These chimeras also showed a higher tendency to form aggregates, similar to GFP-Nup98. Fusions of Nup98 to partners lacking any of these DNA-binding domains, such as GFP-Nup98-LEDGF (Fig 1L) showed no particular distribution within the nucleoplasm. Mutational disruption of the HD (GFP-Nup98-PMX1 N51S, Fig 1M; GFP-Nup98-HOXA9 N51S, Fig 1O; GFP-Nup98-HHEX ΔHD, Fig 1Q), the PHD domain (GFP-Nup98-JARID1A W1625A Fig 1R) or the GLFG domain (GFP-Nup98-HOXA9 ΔFG, Fig 1N; GFP-Nup98-HHEX ΔFG Fig 1P) displaced the Nup98 chimeras from their specific locations. GFP-HOXA9 (Fig 1S) and GFP-HHEX (Fig 1T) were both found diffusely distributed in the nucleoplasm. Together our data indicate that Nup98 chimeras generally localize to the nuclear interior, but their specific intra-nuclear distribution is varying and dependent on the fusion partner, the integrity of the GLFG domain in Nup98 and the integrity of the DNA-binding domains of the respective fusion partner.

**Table 1:** Nup98 fusion proteins employed in this study.

Fusion protein	Fusion type	Reference
Nup98-HOXA9	HD	[23, 25, 56]
Nup98-HOXA10	HD	[57]
Nup98-HHEX	HD	[32]
Nup98-PMX1	HD	[24, 58, 59]
Nup98-JARID1A	non-HD	[60, 61]
Nup98-PHF23	non-HD	[61, 62]
Nup98-NSD1	non-HD	[47, 63–65]
Nup98-NSD3	non-HD	[66, 67]
Nup98-RARG	non-HD	[37, 68]
Nup98-LEDGF	non-HD	[69, 70]

HD, homeo domain.

### Nup98 homeodomain fusion proteins associate with DNA in mitosis

**Fig 2:**
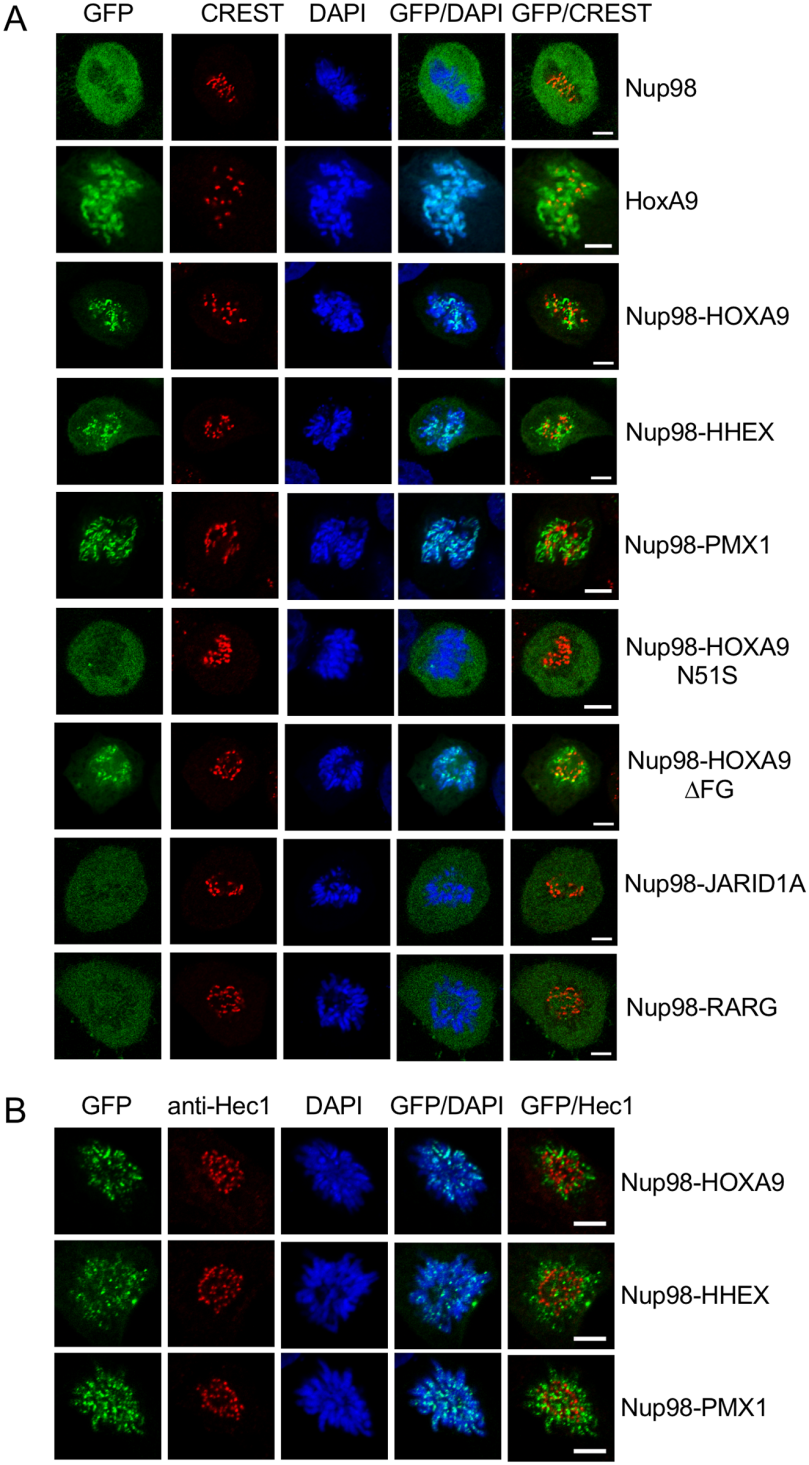
Mitotic localization of Nup98 chimeras. HeLa cells were transiently transfected with GFP constructs and fixed and stained after 24 hours for immunofluorescence microscopy. (**A**) CREST serum, which in particular recognizes CENP-B [36, 55], was used to detect the inner kinetochore and DAPI to visualize DNA. Nup98-HD fusion proteins (Nup98-HOXA9, Nup98-HHEX, Nup98-PMX1; green) associate with chromatin (blue), but not with the inner kinetochore (red) during prometaphase. No association with chromatin was found for Nup98 or Nup98 fused to non-HD partners (i.e. Nup98-JARID1A and Nup98-RARG). Disruption of the HD domain of Nup98-HOXA9 (Nup98-HOXA9 N51S), but not of the FG domain (Nup98-HOXA9 ΔFG) affects chromatin association of the fusion protein. Shown are single confocal sections. Scale bars, 5 μm. (**B**) Anti-Hec1 antibodies were used to detect the outer kinetochore (red), but no co-localization of Nup98-HOXA9, Nup98-HHEX, and Nup98-PMX1, respectively (green) was observed in prometaphase cells. The fusion proteins exclusively associated with chromatin (blue). Shown are single confocal sections. Scale bars, 5 μm.

Nup98-HOXA9, Nup98-HOXD10 and Nup98-PMX1 were previously found to associate with kinetochores and chromosomes during mitosis and it was suggested that they localize to the outer kinetochore [36]. To address whether Nup98 chimeras with non-HD proteins would behave similarly, we transiently transfected HeLa cells with GFP-Nup98, GFP-HOXA9, the HD-fusions GFP-Nup98-HOXA9, GFP-Nup98-HHEX, GFP-Nup98-PMX1, as well as with the non-HD fusions GFP-Nup98-JARID1A and GFP-Nup98-RARG, and stained non-synchronized cells with CREST serum staining the inner kinetochore. As shown in Fig 2A, we found that similar to HOXA9, Nup98-HOXA9, Nup98-HHEX and Nup98-PMX1 were concentrated on chromatin during pro-metaphase (and metaphase; data not shown). The association of the fusion proteins with chromatin was dependent on the integrity of the homeodomain: the HD mutant Nup98-HOXA9 N51S no longer bound chromatin, whereas the Nup98-HOXA9 ΔFG mutant was still found on chromatin. Similar results were obtained for the corresponding Nup98-HHEX and Nup98-PMX1 mutants (data not shown). However, no significant co-localization with the inner kinetochore marker CREST was observed for these Nup98-HD fusion proteins. Furthermore, Nup98-HOXA9, Nup98-HHEX and Nup98-PMX1, respectively, also showed no overlay with the outer kinetochore protein Hec1 [38] during pro-metaphase (Fig 2B) or metaphase (data not shown). In contrast to Nup98-HD fusions and similar to Nup98, the Nup98-JARID1A and Nup98-RARG fusions were not found to be concentrated on chromatin. Together our data show that only Nup98-HD fusion proteins co-localize with chromatin, but not with kinetochores.

### Effects of Nup98 fusion proteins on the nuclear lamina

**Fig 3:**
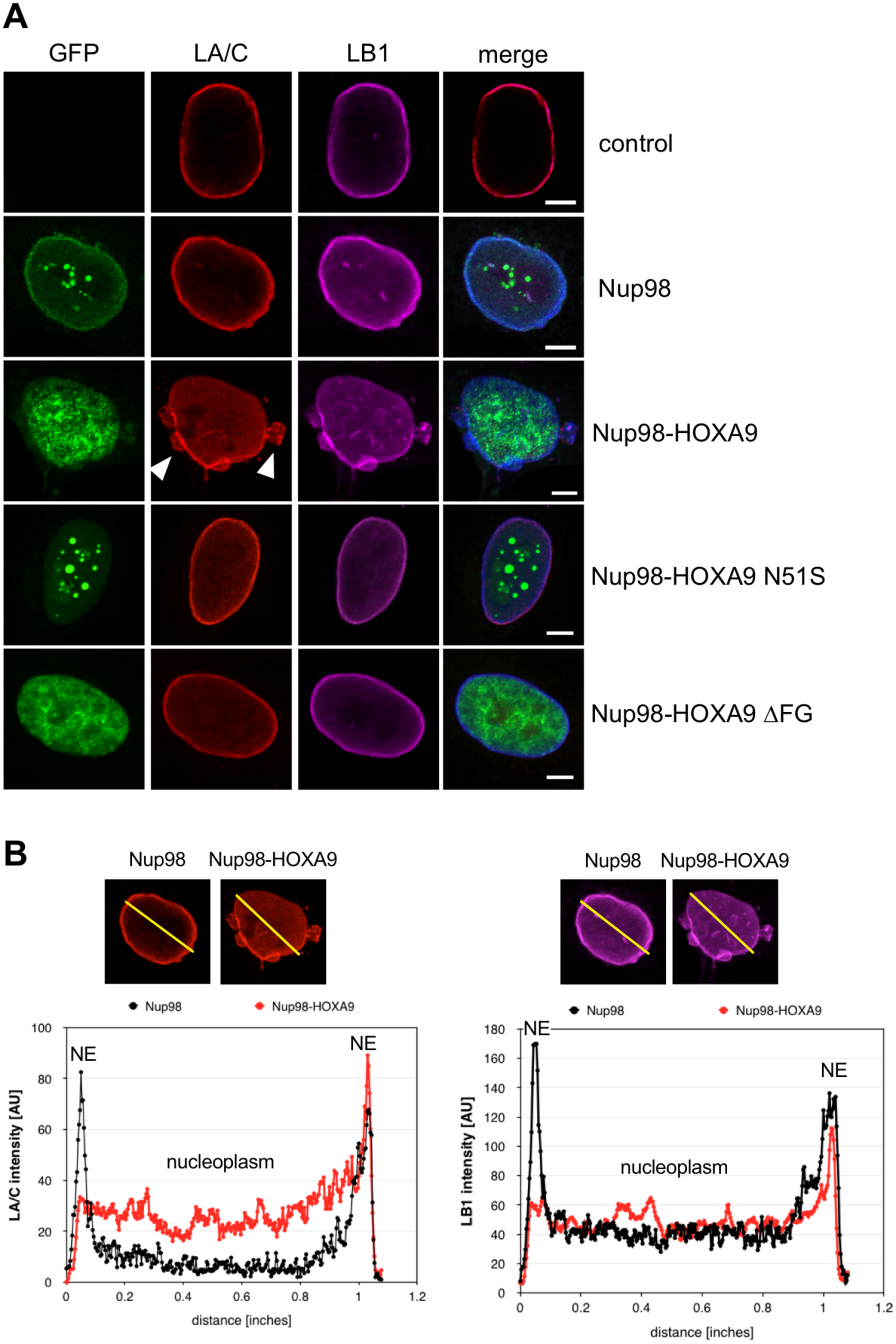
Nup98-HOXA9 affects lamin A/C and lamin B1 distribution. HeLa cells were transiently transfected with GFP constructs and fixed and stained after 24 hours for immunofluorescence microscopy. (**A**) Lamin A/C (LA/C, red) and lamin B1 (LB1, magenta) concentrate at the nuclear envelope (NE) in HeLa cells expressing Nup98 (green), but relocate to the nucleoplasm in cells expressing Nup98-HOXA9. White arrowheads point to some lobules decorating the NE. Disruption of the homeodomain of HOXA9 (Nup98-HOXA9 N51S) and the FG domain of Nup98 (Nup98-HOXA9 ΔFG) prevent the relocation of the lamina proteins. Scale bars, 5 μm. (**B**) Fluorescence intensity of LA/C (left) and LB1 (right) staining was determined along the axis shown as line in the fluorescence images and plotted as a graph.

We next examined the effects of Nup98 fusion protein expression on several components of the NE, i.e. the major constituents of the nuclear lamina, lamin A/C (LA/C) and lamin B1 (LB1) as well as NPCs. NPCs were detected using a monoclonal antibody that recognizes several FG-repeats containing nucleoporins (mAb414). While the organization of the NPC appeared normal (S1 Fig), expression of Nup98 fusion proteins had a strong effect on the nuclear lamina. As shown in Fig 3A, lamin A/C and lamin B1 were found concentrated in the lamina with lower levels in the nucleoplasm of HeLa cells transiently expressing GFP-Nup98 (second row), as well as in untransfected control cells (first row). In contrast, expression of GFP-tagged Nup98 fusion proteins (i.e. Nup98-HOXA9, Nup98-HHEX, Nup98-PMX1, Nup98-NSD1, Nup98-LEDGF) clearly altered the appearance of the lamina: the accumulation of both lamin A/C and lamin B1 in the lamina was reduced, the fraction in the nucleoplasm increased and the NE appeared somehow lobulated (Fig 3A, rows 3–6; S2 Fig). Consistent with this partial displacement of lamins from the lamina other lamina components, such as Sun1, Sun2, and emerin, were partially diminished from the INM, whereas components of the ONM, such as Nesprin-2, remained unaffected (S3 Fig). Intensity profiles across the horizontal axis through nuclei revealed a decrease in the peripheral lamins A/C and B1 along with an increase in the nucleoplasmic lamins A/C and B1 in cells expressing GFP-Nup98-HOXA9 as compared to GFP-Nup98 (Fig 3B). The aberrant NE phenotype was dependent on the integrity of both the HD and the FG domain in Nup98: HeLa cells expressing the HD mutant Nup98-HOXA9 N51S or the Nup98-HOXA9 ΔFG mutant showed lamina staining indistinguishable from cells expressing Nup98 (Fig 3A). Similar results were obtained for the corresponding Nup98-HHEX mutants (data not shown).

**Fig 4:**
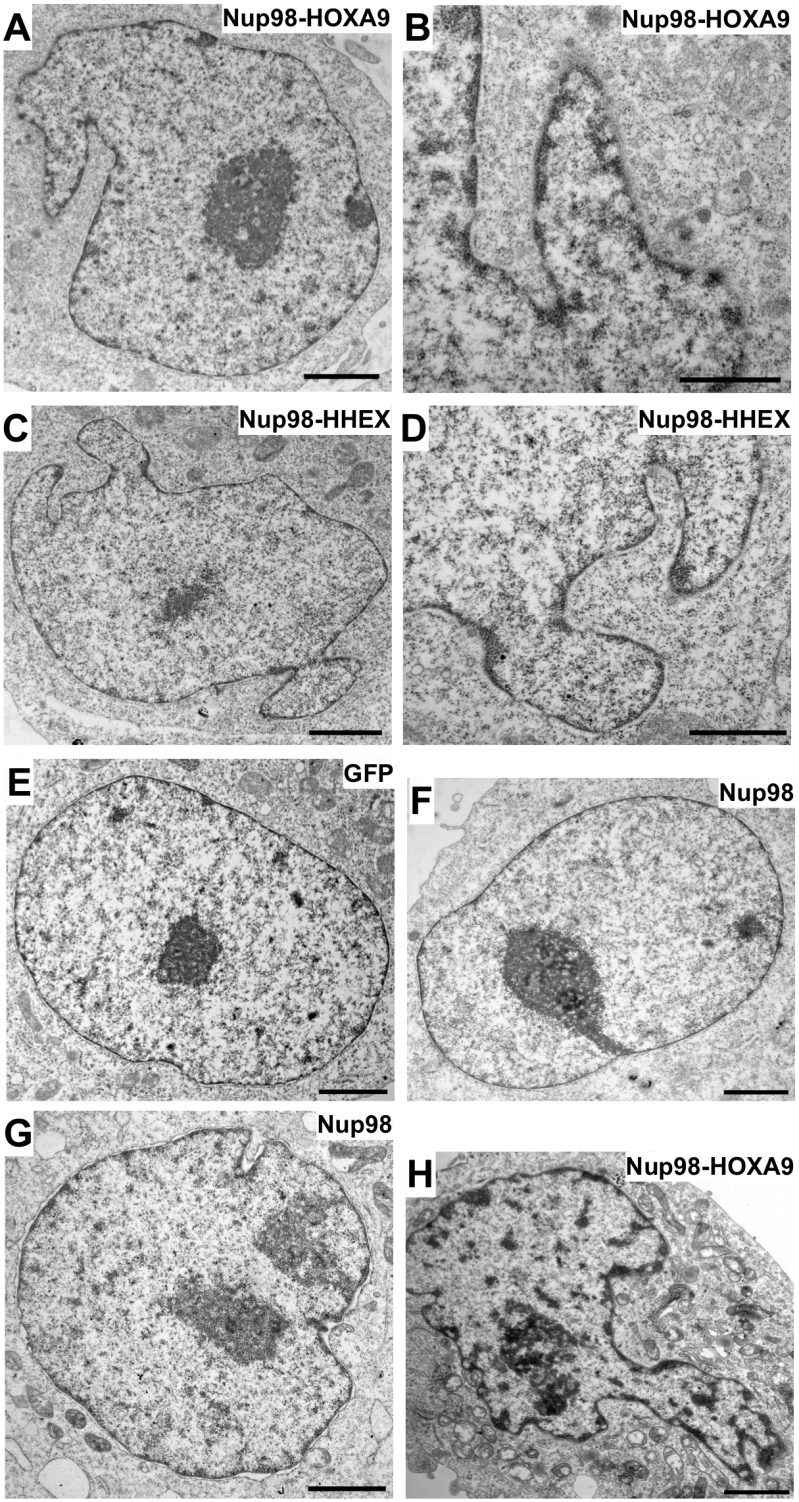
Electron micrographs of HeLa cells expressing Nup98 chimeras. (**A**) and (**B**) Nup98-HOXA9, (**C**) and (**D**) Nup98-HHEX, control cells expressing (**E**) GFP and (**F**) Nup98. HeLa TRex cells expressing (**G**) Nup98 and (**H**) Nup98-HOXA9. Scale bars, 2 μm (A, C, E, F, G, H); 1 μm (B and D).

Electron microscopic imaging of HeLa cells expressing GFP-Nup98-HOXA9 (Fig 4A and 4B) and GFP-Nup98-HHEX (Fig 4C and 4D) clearly revealed nuclear deformation and NE lobulations, which were not observed in GFP (Fig 4E) or GFP-Nup98 (Fig 4F) expressing cells. To further strengthen the specific effect of Nup98-HOXA9 expression on NE organization and nuclear shape, we generated stable HeLa T-Rex cell lines expressing GFP-Nup98-HOXA9 in a tetracycline-inducible manner. Expression levels in clonal cell lines were analyzed by Western blot analysis and protein localization was determined by direct fluorescence microscopy (S4 Fig). At the ultrastructural level, we observed the same alterations in the nuclear shape as well as NE lobulation in HeLa T-Rex cells conditionally expressing GFP-Nup98-HOXA9 (Fig 4H) as in transiently transfected HeLa cells. In the absence of tetracycline and GFP-Nup98-HOXA9 expression, these cells had normal nuclear shape and NE characteristics (Fig 4G).

### Expression of Nup98 fusion proteins results in LAP2α alterations

**Fig 5:**
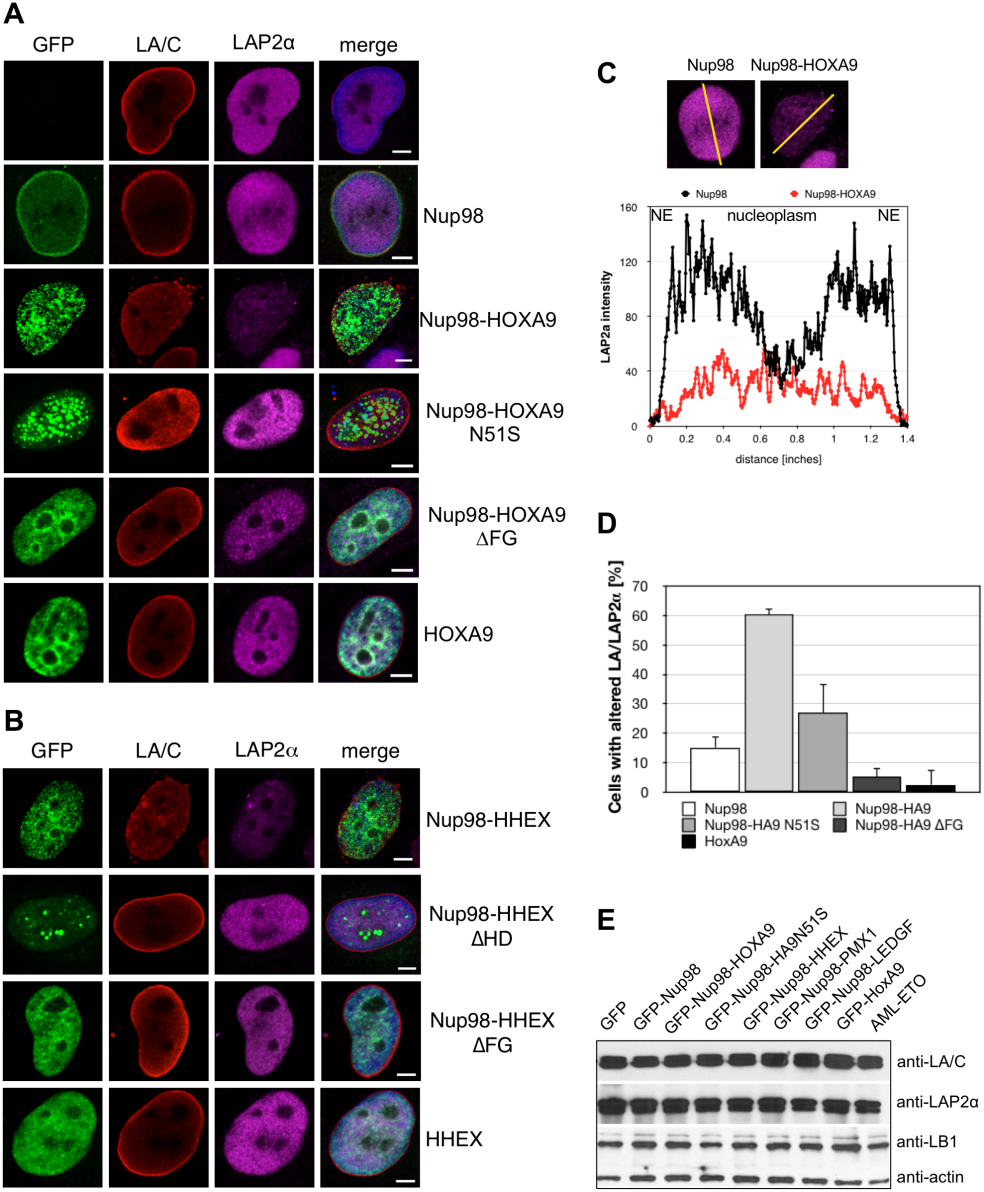
Expression of Nup98 fusions perturbs the nuclear distribution of lamina-associated polypeptide 2α (LAP2α). HeLa cells were transiently transfected with GFP constructs and fixed and stained after 24 hours for immunofluorescence microscopy. (**A**) Lamin A/C (LA/C, red) concentrates at the nuclear envelope in HeLa control cells and in Nup98 expressing cells (green), while LAP2α (magenta) is found throughout the nucleoplasm. In HeLa cells expressing Nup98-HOXA9 (**A**) and Nup98-HHEX (**B**), LAP2α diminished from the nucleoplasm and aggregates at the nuclear periphery. Disruption of the homeodomain in HOXA9 (**A**) and HHEX (**B**) and the FG domain of Nup98 (**A** and **B**) prevent the relocation of the lamina proteins. DAPI was used to visualize DNA (merge). (**B**) Fluorescence intensity of LAP2α staining was determined along the axis shown as line in the fluorescence images and plotted as a graph. (**D**) Quantification of cells with altered LA/C and LAP2α distribution. About 400 cells were analyzed for each sample. (**E**) Western blot analysis of the expression levels of LA/C, LAP2α, and LB1.

We next asked whether nucleoplasmic lamin-binding proteins, such as the lamin A-binding partner LAP2α, might be affected by Nup98 chimeras [39]. As shown in Fig 5A (top row), LAP2α is a nucleoplasmic protein excluded from the nucleoli, in contrast to the primarily lamina-associated lamin A/C. Expression of GFP-Nup98 (Fig 5A, second row) did not alter the distribution of lamin A/C or LAP2α. In contrast, cells expressing GFP-Nup98-HOXA9 (Fig 5A, third row) or GFP-Nup98-HHEX (Fig 5B, first row) exhibited a dramatically reduced LAP2α staining in the nucleoplasm and a frequent aggregation of LAP2α in small foci at the nuclear periphery in addition to the alterations in lamin A/C. Plotting LAP2α staining intensity profiles across the nuclear diameter confirmed this decrease in LAP2α staining throughout the nucleoplasm (Fig 5C). GFP-HOXA9 (Fig 5A, bottom row) or GFP-HHEX (Fig 5B, bottom row) expression had no effect on the distribution of the two proteins. In contrast, the changes in lamin A/C and LAP2α distribution were also found in HeLa cells expressing GFP-Nup98-PMX1 or the non-HD fusion proteins GFP-Nup98-NSD1 and GFP-Nup98-NSD3 (S5A Fig), but were not seen in cells expressing other AML-related chromosomal translocation products, such as the AML1-ETO fusion protein (S5B Fig).

The specificity of the effect on lamin A/C and LAP2α was further supported by the observation that expression of fusions with either disrupted homeodomain of HOXA9 (Nup98-HA9 N51S; Fig 5A, forth row) or HHEX (Nup98-HHEX ΔHD; Fig 5B, second row) or disruption of the FG domain in Nup98 (Nup98-HA9 ΔFG; Fig 5A, fifth row and Nup98-HHEX ΔFG; Fig 5B, third row) did not result in the lamin A/C and LAP2α alterations. Quantification of our observations (Fig 5D) revealed that in about 60% of Nup98-HOXA9 expressing cells the distribution of lamin A/C and LAP2α was altered, in contrast to about 15% in cells expressing Nup98 and about 3% of HOXA9 expressing cells. In cells expressing Nup98-HOXA9 N51S we found about 28% of the cells having alterations in lamin A/C and LAP2α localization and upon Nup98-HOXA9 ΔFG expression only 8% of the cells had perturbed lamin A/C and LAP2α pattern. The effect on the intranuclear pattern of lamin A/C and LAP2α did neither change expression levels of lamin A/C and LAP2α as well as lamin B1 (Fig 5E) nor lamin A processing (S5C Fig) or the physical association of lamin A/C with LAP2α (data not shown). Together our data indicate that Nup98 fusion proteins specifically affect the intranuclear distribution of lamin A/C and its binding partner LAP2α without affecting lamin A/C expression levels or processing.

### Altered LAP2α distribution in leukemic cells carrying Nup98 fusions

**Fig 6:**
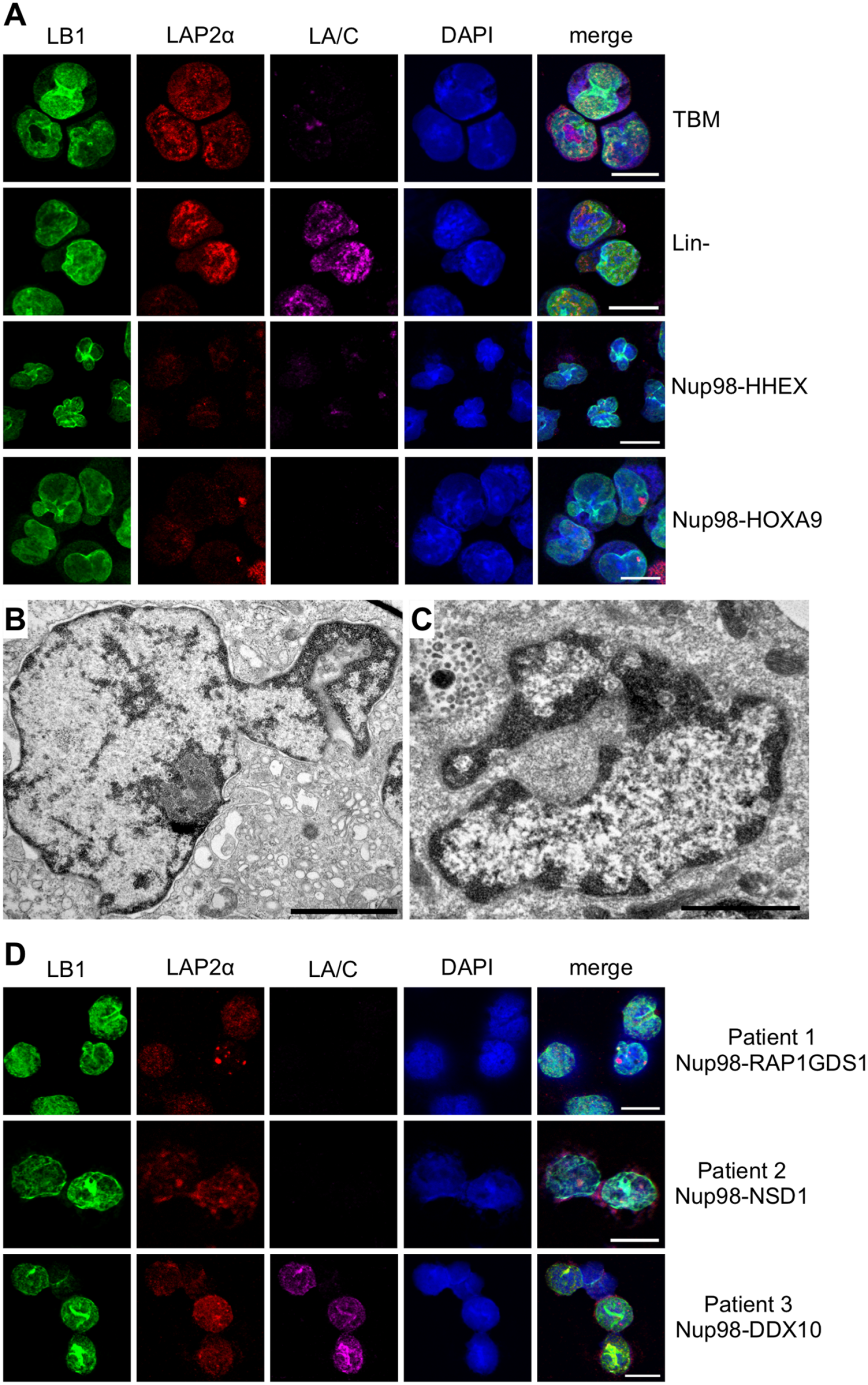
Lamina-associated polypeptide 2α (LAP2α) is altered in Nup98 fusion expressing leukemic cells. (**A**) Mouse bone marrow cells were transduced with retroviral particles to express untagged Nup98-HOXA9 and Nup98-HHEX and stained for immunofluorescence microscopy. Expression of these fusion proteins induced lobulations in the NE as evident from the lamin B1 staining (LB1, green). Whereas lamin A/C is not expressed in mouse BM cells (LA/C, magenta), LAP2α (red) is evenly distributed in nuclei from total bone marrow (TBM) cells and lineage minus precursor cells (Lin-), it is diminished from the nucleoplasm and aggregates at the nuclear periphery of mouse BM cells expressing Nup98-HHEX and Nup98-HOXA9, respectively. Electron micrographs of mouse bone marrow cells transduced with (**B**) Nup98-HHEX and (**C**) Nup98-HOXA9. Scale bars, 2 μm (C); 1 μm (B). (**D**) Lamin B1 staining of patient-derived bone marrow cells revealed irregular NE contour. Lamin A/C is not consistently expressed in patient cells and LAP2α is diminished from the nucleoplasm and aggregates at the nuclear periphery. Patient 1 harbored a Nup98-RAP1GDS1, patient 2 a Nup98-NSD1, and patient 3 a Nup98-DDX10 fusion, respectively. Scale bars, 10 μm.

Next we asked if the aberrant NE phenotype could also be observed in leukemic cells carrying Nup98 fusions. Lacking established human leukemia cell lines expressing Nup98 fusions we first immortalized mouse bone marrow (BM) cells by retroviral expression of untagged Nup98-HOXA9 and Nup98-HHEX. The cells were expanded by serial plating and immortalized cell lines were established [32]. As shown in Fig 6A and S6 Fig, Nup98-HOXA9 and Nup98-HHEX expressing mouse BM cells, similar to normal total bone marrow (TBM) cells and lineage marker-depleted (Lin-) BM progenitor cells, expressed no or only very small amounts of lamin A/C, in contrast to the BM-derived B cell line Ba/F3. Lamin B1 staining revealed that Nup98-HOXA9 and Nup98-HHEX expressing cells have strongly lobulated nuclei in contrast to TBM and Lin- cells, with a slight increase in the nucleoplasmic pool of lamin B1 (Fig 6A, first row). These NE lobulations were also readily detectable on electron microscopic level (Fig 6B and 6C). In TBM and Lin- BM cells LAP2α localized to the nucleoplasm, whereas Nup98-HOXA9 and Nup98-HHEX expressing cells showed only weak labeling and aggregation of LAP2α. The expression levels of LAP2α, lamin A/C and lamin B1 on mRNA level were similar in all cell types (S6 Fig). We also analyzed lamin B1, lamin A/C and LAP2α localization in bone marrow derived leukemic blasts of three patients with Nup98 alterations (S3 Table). As shown in Fig 6D, only one of the patients (patient 3 with *NUP98-DDX10*) expressed some lamin A/C, while the other two did not (patient 1 with *NUP98-RAP1GDS1*, patient 2 with *NUP98-NSD1*). Lamin B1 localized to the NE and the nucleoplasm revealing nuclear lobulation, whereas LAP2α was barely detectable and often aggregated, indicating that alterations in LAP2α localization also occur in leukemic blasts with *NUP98* translocations. Collectively these data indicate that the aberrant NE phenotype observed in transfected HeLa cells is also found in murine bone marrow cells immortalized by Nup98 fusions as well as in primary tumor cells from leukemia patients carrying translocations leading to Nup98 fusions.

### Presence of Nup98 chimeras altered cell cycle progression

**Fig 7:**
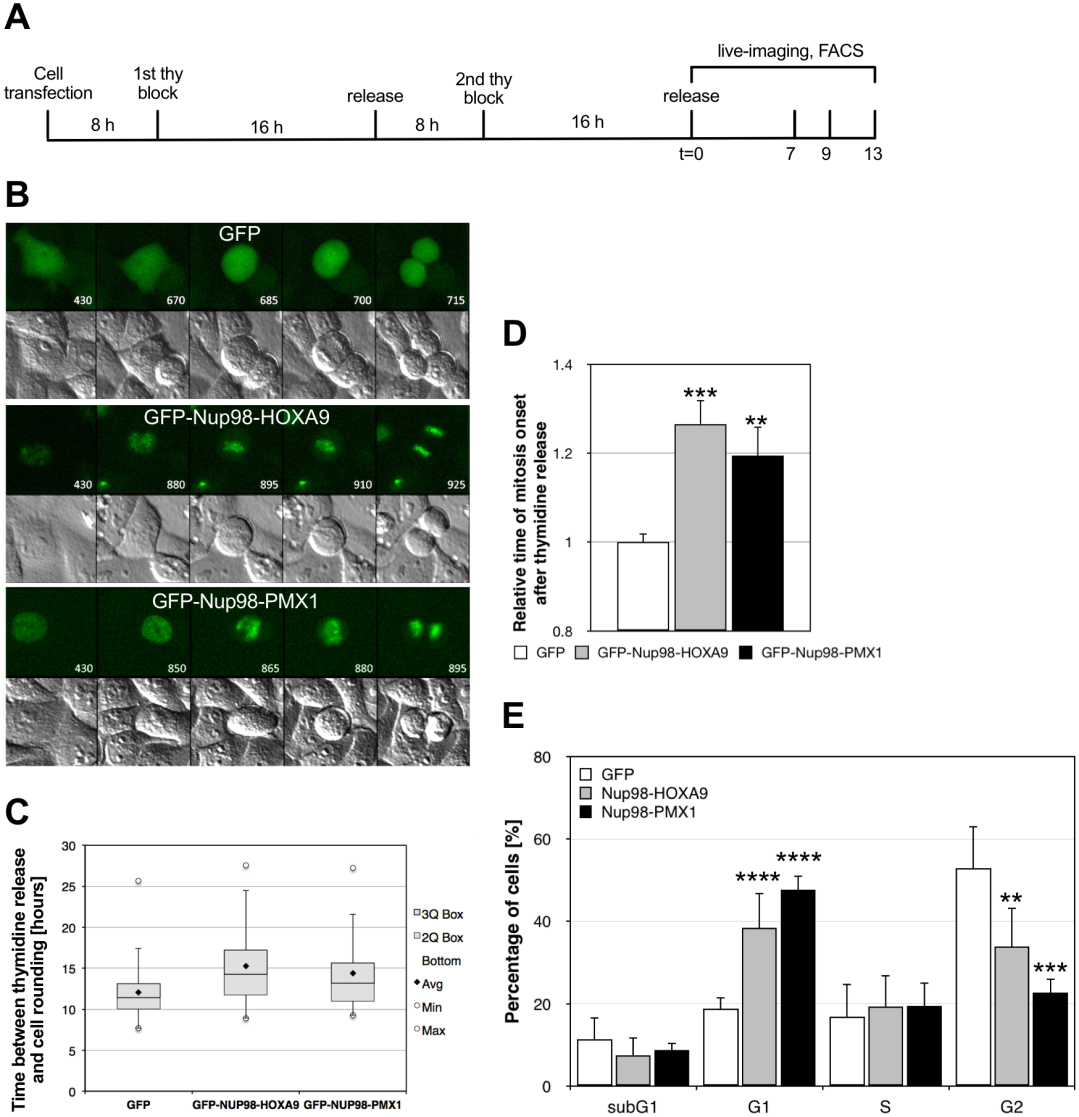
Expression of Nup98 fusion proteins deregulates cell cycle progression. HeLa cells were transiently transfected with GFP-Nup98-HOXA9 and GFP-Nup98-PMX1 and analyzed by live cell imaging and flow cytometry after release from a double-thymidine block. (**A**) Schematic presentation of the time course for live cell imaging and flow cytometric analysis. (**B**) GFP and differential interference contrast time-lapse images of HeLa cells after release from double-thymidine block are presented. Cells expressing Nup98-HOXA9 and Nup98-PMX1, respectively, show a delay in the re-entry into the cell cycle. Time is indicated in minutes. (**C**) Boxplot display of the quantitative analysis of the time between double-thymidine release and onset of mitosis. (**D**) Quantitative analysis of the relative time of mitosis onset. The differences between GFP, GFP-Nup98-HOXA9 and GFP-Nup98-PMX1 expressing cells were statistically highly relevant (**P>0.01; ***P>0.001). (**E**) DNA flow cytometry of control, GFP-Nup98-HOXA9 and GFP-Nup98-PMX1 expressing cells nine hours after release a double thymidine block. The differences between GFP, GFP-Nup98-HOXA9 and GFP-Nup98-PMX1 expressing cells were statistically highly relevant (**P>0.01; ***P>0.001; ****P>0.0001).

LAP2α is known to regulate cell cycle progression and differentiation via the retinoblastoma-E2F pathway [40, 41]. Overexpression of LAP2α has been shown to reduce G1/S transition, whereas knock down of LAP2α enhanced cell cycle progression [40, 41]. We therefore asked whether Nup98 fusion expression might affect cell cycle progression. We carried out time lapse imaging of live cells expressing Nup98-HOXA9 and Nup98-PMX1, respectively, in combination with flow cytometric analyses (FACS) of the cell cycle. HeLa cells were transfected and subjected to a double thymidine block 8 hours after transfection (Fig 7A). A double thymidine block arrests cells at the G1/S border and cells will enter S phase after release into fresh medium and progress further in the cell cycle [42]. Live imaging was started 7–8 hours after release from double thymidine (Fig 7B, 430 minutes) and HeLa cells expressing GFP entered mitosis about 11 hours after the release (Fig 7B; top two rows, 670 minutes; Fig 6C) and progressed through mitosis within 45 minutes. HeLa cells expressing GFP-Nup98-HOXA9 and GFP-Nup98-PMX1 showed a significant delayed entry into mitosis at only about 15 hours after the release from double thymidine block (Fig 7B; 880 and 850 minutes; Fig 7C). Progression through mitosis in both GFP-Nup98-HOXA9 and GFP-Nup98-PMX1 expressing cells took about 45 minutes, similar to cells expressing GFP. Consistent with the time-lapse microscopy, FACS analyses of re-entry into the cycle revealed that Nup98-HOXA9 and Nup98-PMX1 expressing cells exhibited a delay in S phase onset. Double thymidine treatment arrested about 70% of the differently transfected HeLa cells in G1 (T0, Table 2 and S7A Fig). Nine hours after release from thymidine, 53% of GFP-expressing HeLa cells have reached G2, but only about 34% of Nup98-HOXA9 and about 23% of Nup98-PMX1 expressing cells (Fig 7E, Table 2), indicating a delay in G1/S transition. 13 hours after release it is not further possible to distinguish between cells that have passed mitosis and those delayed in cell cycle progression (T13, Table 2 and S7B Fig).

**Table 2:** Cell cycle analysis of transiently transfected HeLa cells after double thymidine block.

	subG1	G1	S	G2	time after release [hours]
**GFP**	11.4 ± 6.8	68.0 ± 12.2	14.8 ± 5.0	5.1 ± 2.3	0
**Nup98-HOXA9**	8.0 ± 2.2	71.8 ± 3.7	14.7 ± 2.5	4.7 ± 1.3	0
**Nup98-PMX1**	10.6 ± 2.5	68.8 ± 6.3	15.4 ± 4.2	4.5 ± 1.0	0
**GFP**	11.3 ± 5.4	18.9 ± 2.6	16.8 ± 8.1	53.0 ± 10.2	9
**Nup98-HOXA9**	7.5 ± 4.3	38.5 ± 8.3	19.2 ± 7.7	34.0 ± 9.4	9
**Nup98-PMX1**	8.7 ± 1.8	47.7 ± 3.4	19.4 ± 5.6	22.7 ± 3.4	9
**GFP**	16.2 ±13.9	44.7 ± 14.9	10.7 ±4.3	28.0 ± 10.8	13
**Nup98-HOXA9**	12.6 ± 8.0	46.2 ± 7.0	14.6 ± 4.6	25.4 ± 6.0	13
**Nup98-PMX1**	22.7 ± 8.8	38.1 ± 5.4	14.7 ± 1.8	23.6 ± 6.7	13

## Discussion

Previous studies suggested that Nup98-HD fusion proteins adopt a particular intra-nuclear localization and that the chimeras could associate with chromatin and the outer kinetochores [23, 24, 32, 36]. By carrying out a more systematic analysis of Nup98 fusions we show that Nup98 fusions with a homeodomain clearly differ in their intra-nuclear localization from Nup98 fusion proteins without a HD. Likewise, we also found no consistent localization of Nup98 chimeras during mitosis. However, we observed that Nup98 chimeras uniformly provoked morphological alterations of the NE with particular changes in the nuclear lamina and LAP2α that coincided with defects in cell cycle progression. Alterations in the NE and LAP2α were homogenously found in HeLa and mouse BM cells immortalized by Nup98 fusion proteins as well as in patient-derived cells, suggesting that NE alterations are linked to Nup98 fusion-mediated transformation.

We observed that the intra-nuclear localization of Nup98 fusions is not uniform: Nup98 fusions with HD proteins adopt a similar finely speckled localization pattern (Fig 1B–1E), which is consistent with previous studies [23, 24, 32, 36]. Nup98 chimeras with other chromatin-binding proteins, such as histone methyltransferases NSD1and NSD3 and the histone demethylase JARID1A, show a finer punctate intra-nuclear distribution (Fig 1F–1K; see also [37]), which can be clearly distinguished from Nup98-HD fusions. The particular intra-nuclear localization of the Nup98 fusions is significantly perturbed by disruption of the HD domain or the PHD finger in the partner protein, as well as FG domain in Nup98 (Fig 1N–1R).

Similar to HOXA9, Nup98-HD fusion proteins bind to chromatin during mitosis, in contrast to non-HD chimeras, such as Nup98-JARID1A and Nup98-RARG (Fig 2A). Therefore, we conclude that chromatin association during mitosis is not a general feature of Nup98 fusion proteins. The association with chromatin can be attributed to the HD, as its disruption displaces the fusion protein from DNA (Fig 2A, Nup98-HOXA9 N51S). A mutant FG domain in Nup98 had in contrast no effect on the mitotic localization of Nup98-HOXA9 (Fig 2A, Nup98-HOXA9 ΔFG). Interestingly localization of the global *HOX* gene regulator mixed lineage leukemia 1 (MLL) was previously also assigned to a similar nuclear punctate localization [43]. MLL was also found to associate with mitotic chromatin and multiple MLL-dependent *HOX* genes displayed MLL occupancy in both interphase and mitosis [44]. More recently Nup98 was found to physically interact with MLL and to be essential for *HOX* gene expression in *Drosophila* cells [45]. Whether mitotic association of Nup98-HD fusions depends on MLL remains to be investigated.

Xu and Powers found no co-localization of Nup98 chimeras with the CREST marker and concluded indirectly that they therefore bind to outer kinetochores [36]. Using markers for the inner and outer kinetochores, CREST and Hec1, we found no evidence for association of the Nup98-HD fusion proteins with kinetochores [36]. Altogether our results show that Nup98 chimeras adopt heterogeneous cellular localizations during interphase and mitosis. As *in vitro* and *in vivo* transforming activities have been demonstrated for both, Nup98-HD and Nup98-non-HD fusions [26, 31, 32, 46, 47], our work suggests that cellular localization might not be determining their leukemogenic potential.

Expression of Nup98 fusion proteins uniformly provoked an aberrant NE phenotype with nuclear lobulations, relocalization of A- and B-type lamins (Fig 3 and S2 Fig) and alterations in the lamin A-binding partner LAP2α (Figs 5, 6 and S5 Fig). Importantly, this exceptional NE phenotype was not only found in HeLa cells expressing the Nup98 fusion proteins, but also in Nup98 fusion immortalized mouse bone marrow cells and in leukemic blasts of three patients carrying Nup98 fusions. Nuclear lobulation is likely a consequence of altered chromatin organization as the nuclei of Nup98 fusion expressing HeLa cells and mouse bone marrow cells were often enriched in heterochromatin, in particular in the lobules (Fig 4). Changes in chromatin structure might arise from the alterations in LAP2α, which is known to bind to the nucleosome-binding protein high mobility group protein N 5 (HMGN5), and HMNG5 and LAP2α reciprocally affect their genome-wide chromatin distribution [48]. Our work suggests a novel role for LAP2α in chromatin and NE organization associated with malignant transformation that needs further investigations.

The lamin A/C- LAP2α complex is known to regulate cell cycle progression and differentiation via the retinoblastoma (Rb)-E2F pathway [39–41]. Consistent with alterations in the Rb-E2F pathway, a slowed progression from G1 into S phase was observed in HeLa cells expressing Nup98 fusions proteins after a double thymidine arrest (Fig 7). This is similar to what had previously been described for LAP2α overexpression [40, 49]. However, Western blot and qRT-PCR analyses revealed no changes in LAP2α levels in Nup98 fusion-expressing HeLa and mouse bone marrow cells (Fig 5 and S5 Fig), while our imaging approaches consistently showed a reduced intensity of LAP2α along with some aggregation of the protein. The reduced intensity could be due to epitope masking of the LAP2α antibodies, likely due to a posttranslational modification or to conformational change in LAP2α. According to the crystal structure of the C-terminal coiled-coil domain, LAP2α can form dimers [50] or even trimers [51]. Therefore, in cells expressing Nup98 chimeras, LAP2α might form other oligomeric structures due to presence or absence of a yet to identify binding partner. This change in the oligomeric state of LAP2α may interfere with the epitopes of the two anti- LAP2α antibodies employed for our immunofluorescence analyses: both recognize the C terminus of LAP2α. Binding to Rb on the contrary may not be affected or even stabilized, which would have a similar effect than overexpression of LAP2α. Further biochemical characterization of the LAP2α isolated from cells expressing Nup98 chimeras is necessary to precisely understand their effects on LAP2α.

Taken together, we have shown that while Nup98 fusion proteins lack a uniform localization during interphase and mitosis, they consistently provoke alterations in the NE and LAP2α, which coincide with defects in cell cycle progression. Our study supports a functional significance of LAP2α in cancer, although its specific role remains to be elucidated. The role of LAP2α in Nup98-associated leukemia is likely independent of lamin A/C as mouse bone marrow cells and patient-derived bone marrow cells show alterations in LAP2α in a manner independent of lamin A/C expression (Fig 6). LAP2α binds Rb and lamin A/C independently [39, 40] and it is important for the development of normal hematopoiesis [41, 52–54]. How this is connected to our observations is subject of ongoing investigations.

## Supporting Information

S1 Fig Nuclear pore complex distribution is unaffected in the presence of Nup98 chimeras. HeLa cells were transiently transfected with GFP constructs and fixed and stained after 24 hours for immunofluorescence microscopy. A monoclonal antibody (mAb414) was used to detect nuclear pore complexes (red). Scale bars, 5 μm.
(PDF)

S2 Fig Nup98 fusion proteins affect lamin A/C and lamin B1 distribution. HeLa cells were transiently transfected with GFP constructs and fixed and stained after 24 hours for immunofluorescence microscopy. (**A**) Lamin A/C (LA/C, red) and lamin B1 (LB1, magenta) relocate to the nucleoplasm in cells expressing Nup98-PMX1, Nup98-HHEX, Nup98-NSD1, and to a lesser extend Nup98-LEDGF, respectively. White arrowheads point to some lobules decorating the NE. Scale bars, 5 μm.
(PDF)

S3 Fig Nup98 fusion proteins affect inner nuclear membrane proteins. HeLa cells were transiently transfected with GFP constructs and fixed and stained after 24 hours for immunofluorescence microscopy. In comparison to Nup98 expressing HeLa cells, the inner nuclear membrane proteins (**A**) Sun1, (**B**) Sun2, and (**C**) emerin are reduced at the nuclear envelope in cells expressing Nup98-HOXA9, Nup98-JARID1A, and Nup98-RARG, respectively, but not so the outer nuclear membrane protein Nesprin-2 (**D**). Scale bars, 5 μm.
(PDF)

S4 Fig HeLa TRex cells expressing GFP-Nup98 and GFP-Nup98-HOXA9, respectively, upon treatment with tetracycline for 24 hours. (**A**) Immunofluorescence microscopy revealed the correct localization of the GFP-tagged proteins during interphase and mitosis. Scale bars; 10 μm, upper and middle row; 5 μm lower row. (**B**) Western blot analysis of three selected clones to determine the relative expression of the GFP-tagged proteins for each clone. Proteins were detected with an anti-GFP antibody.
(PDF)

S5 Fig Oncogenic Nup98 fusion proteins perturb the nuclear distribution of lamina-associated polypeptide 2α (LAP2α). HeLa cells were transiently transfected with GFP constructs and fixed and stained after 24 hours for immunofluorescence microscopy. (**A**) In HeLa cells expressing Nup98-PMX1, Nup98-NSD1, and Nup98-NSD3, respectively, LAP2α is diminished from the nucleoplasm and aggregates at the nuclear periphery. (**B**) Lamin A/C (LA/C, red; top row) concentrates at the nuclear envelope in HeLa expressing AML1-ETO, while LAP2α (red; bottom row) is found throughout the nucleoplasm. Scale bars, 5 μm. (**C**) Western blot analysis of the expression levels of LA/C, pre-lamin (pre-LA), farnesylated pre-LA, and progerin in HeLa cells and HeLa cells expressing GFP-Nup98, GFP-Nup98-HOXA9, GFP-Nup98-JARID1A, respectively. Actin was used as loading control.
(PDF)

S6 Fig Western blot and qRT-PCR analysis to determine the relative expression of lamin B, lamin A and LAP2α, respectively in non-transformed and transformed mouse bone marrow cells. (PDF)

S7 Fig DNA flow cytometry of control, GFP-Nup98-HOXA9, and GFP-Nup98-PMX1 expressing cells (A) directly after release from a double thymidine block and (B) 13 hours after release into fresh medium. (PDF)

S1 Table Plasmids used in this study. (DOCX)

S2 Table qRT-PCR Primer. (DOCX)

S3 Table Hematological and cytogenetic features of patient samples. AML, acute myeloid leukemia; RAEB, refractory anemia with excess of blast; T-ALL, T-cell acute lymphoblastic leukemia.
(DOCX)
