# Supplementary figures and images for: Expression of Leukemia-Associated Nup98 Fusion Proteins Generates an Aberrant Nuclear Envelope Phenotype
Source: PLoS One. 2016 Mar 31;11(3):e0152321. doi: 10.1371/journal.pone.0152321 (PMC4816316; doi:10.1371/journal.pone.0152321)

GFP

mAb414

merge

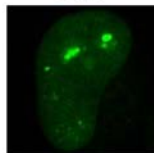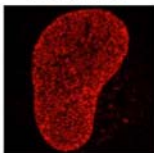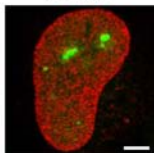

Nup98

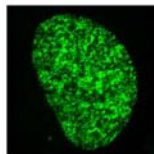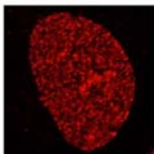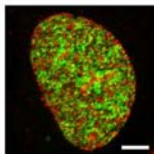

Nup98-HOXA9

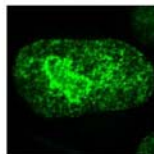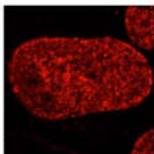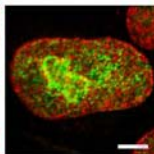

Nup98-HHEX

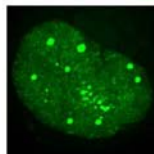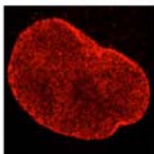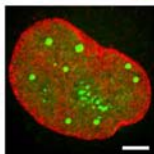

Nup98-NSD1

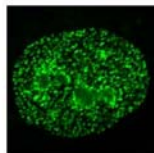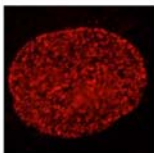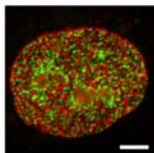

Nup98-PMX1

Supplement: S1 Fig — HeLa cells were transiently transfected with GFP constructs and fixed and stained after 24 hours for immunofluorescence microscopy. A monoclonal antibody (mAb414) was used to detect nuclear pore complexes (red). Scale bars, 5 μm. (PDF) [file pone.0152321.s001.pdf]

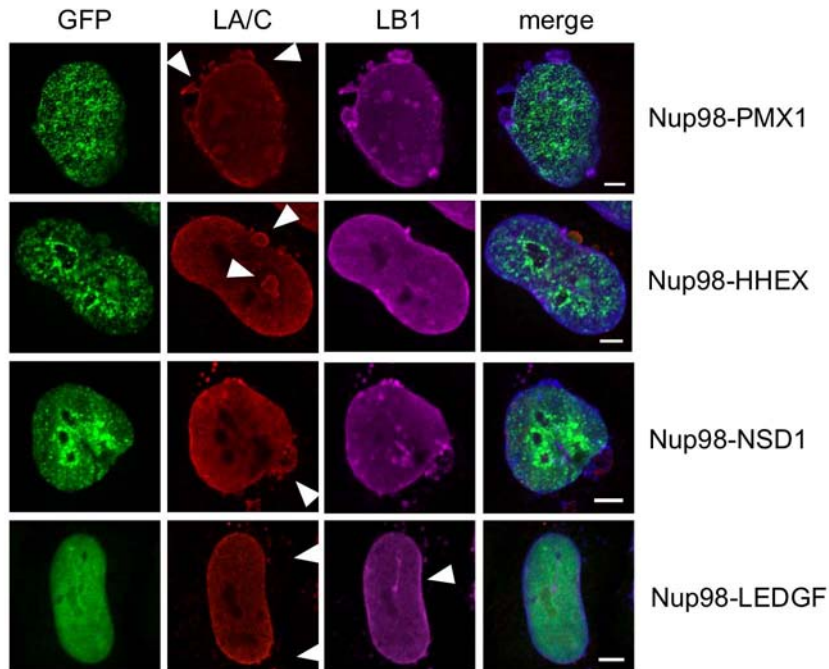

Supplement: S2 Fig — HeLa cells were transiently transfected with GFP constructs and fixed and stained after 24 hours for immunofluorescence microscopy. (A) Lamin A/C (LA/C, red) and lamin B1 (LB1, magenta) relocate to the nucleoplasm in cells expressing Nup98-PMX1, Nup98-HHEX, Nup98-NSD1, and to a lesser extend Nup98-LEDGF, respectively. White arrowheads point to some lobules decorating the NE. Scale bars, 5 μm. (PDF) [file pone.0152321.s002.pdf]

**A**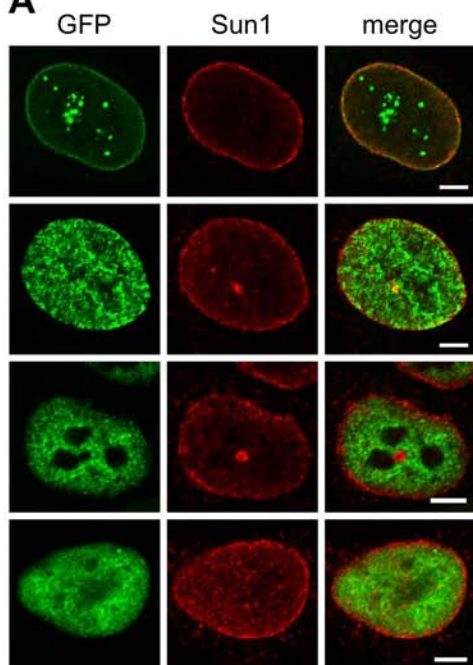**B**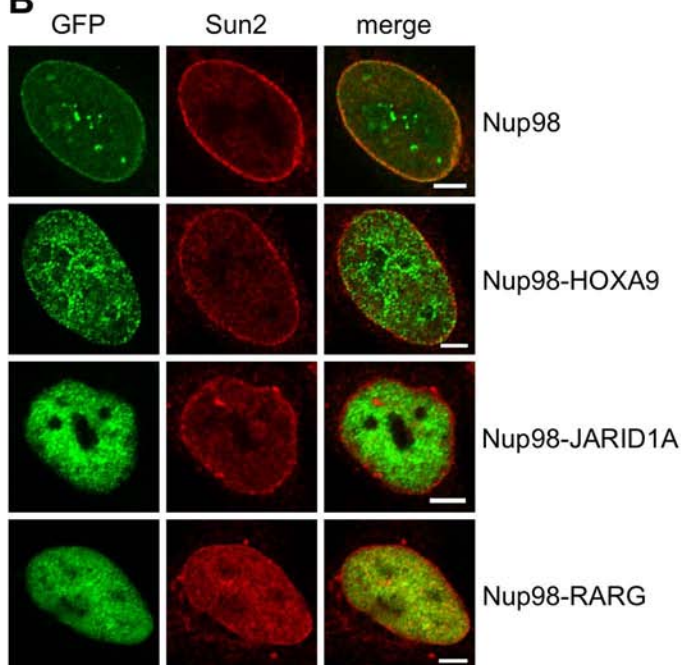**C**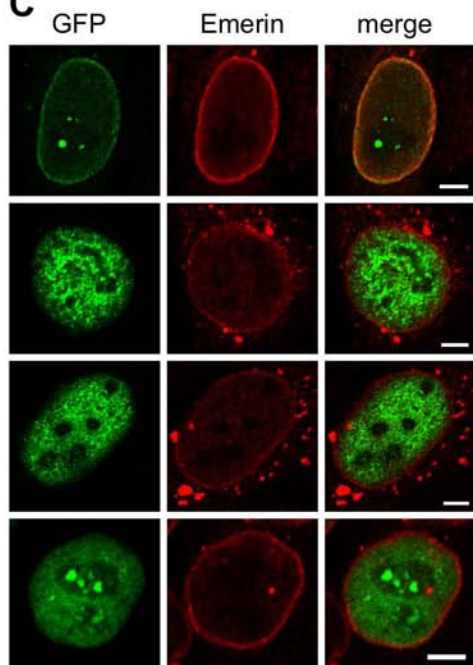**D**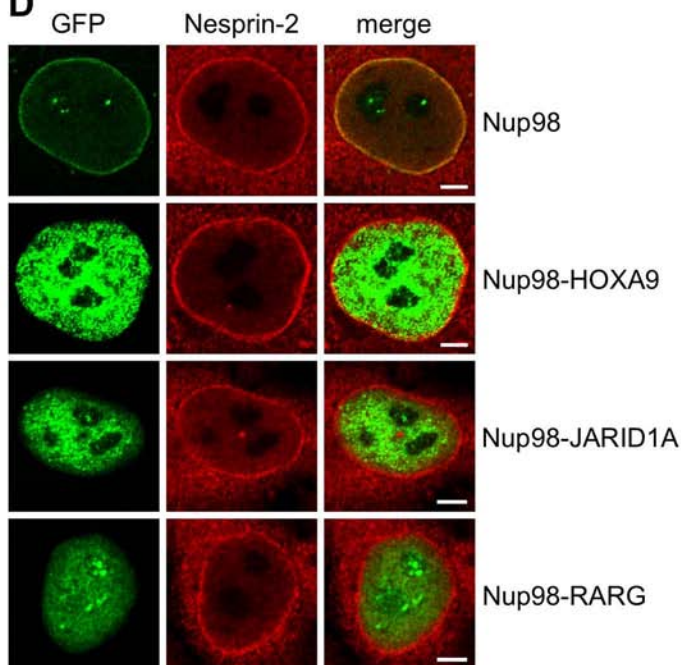

Supplement: S3 Fig — HeLa cells were transiently transfected with GFP constructs and fixed and stained after 24 hours for immunofluorescence microscopy. In comparison to Nup98 expressing HeLa cells, the inner nuclear membrane proteins (A) Sun1, (B) Sun2, and (C) emerin are reduced at the nuclear envelope in cells expressing Nup98-HOXA9, Nup98-JARID1A, and Nup98-RARG, respectively, but not so the outer nuclear membrane protein Nesprin-2 (D). Scale bars, 5 μm. (PDF) [file pone.0152321.s003.pdf]

**A**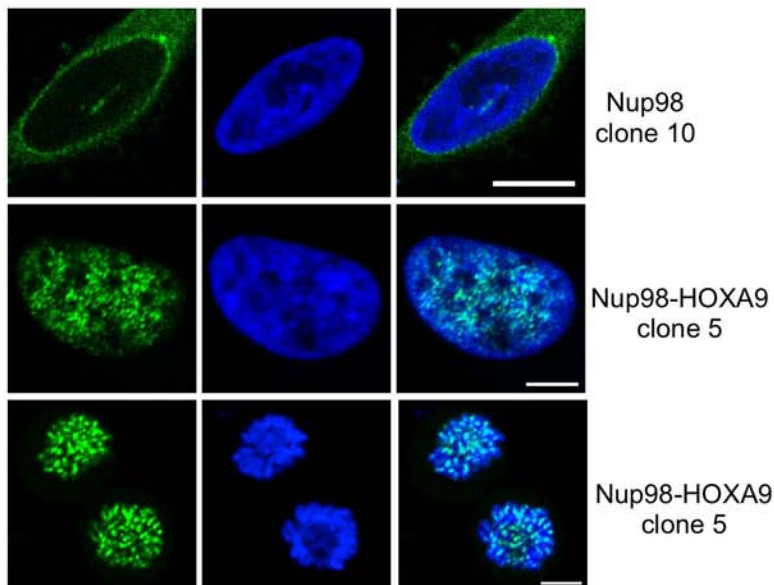**B**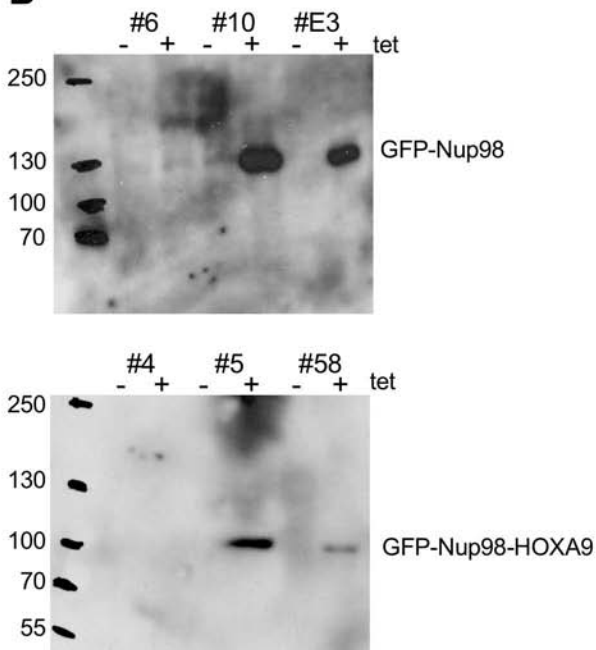

Supplement: S4 Fig — (A) Immunofluorescence microscopy revealed the correct localization of the GFP-tagged proteins during interphase and mitosis. Scale bars; 10 μm, upper and middle row; 5 μm lower row. (B) Western blot analysis of three selected clones to determine the relative expression of the GFP-tagged proteins for each clone. Proteins were detected with an anti-GFP antibody. (PDF) [file pone.0152321.s004.pdf]

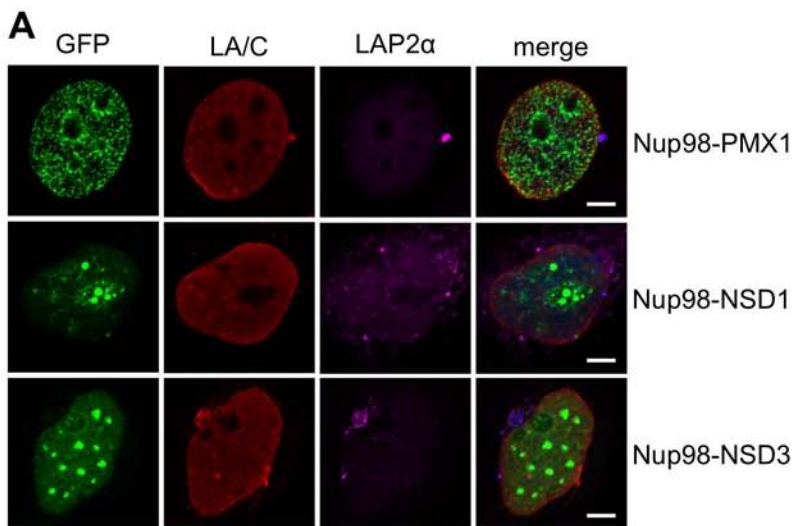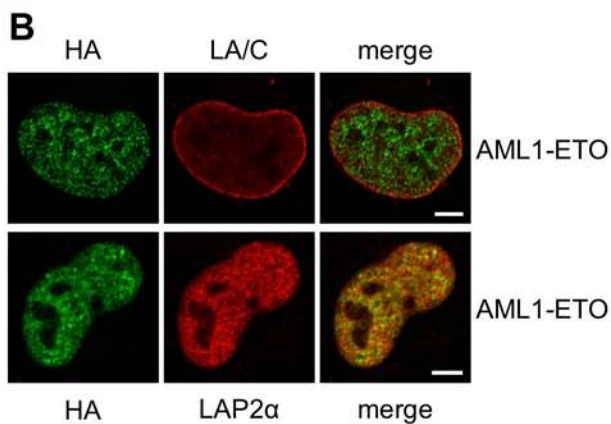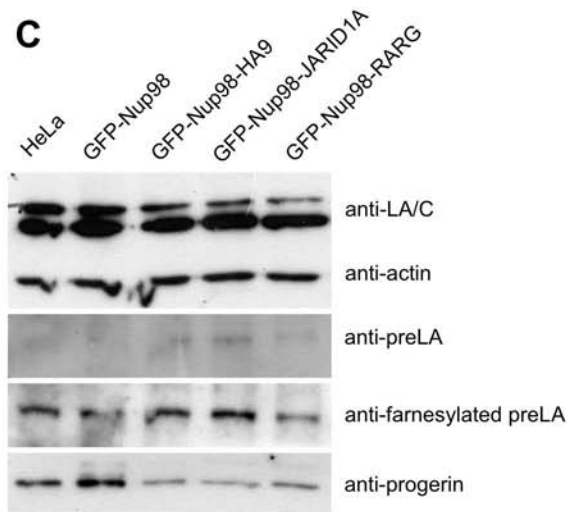

Supplement: S5 Fig — HeLa cells were transiently transfected with GFP constructs and fixed and stained after 24 hours for immunofluorescence microscopy. (A) In HeLa cells expressing Nup98-PMX1, Nup98-NSD1, and Nup98-NSD3, respectively, LAP2α is diminished from the nucleoplasm and aggregates at the nuclear periphery. (B) Lamin A/C (LA/C, red; top row) concentrates at the nuclear envelope in HeLa expressing AML1-ETO, while LAP2α (red; bottom row) is found throughout the nucleoplasm. Scale bars, 5 μm. (C) Western blot analysis of the expression levels of LA/C, pre-lamin (pre-LA), farnesylated pre-LA, and progerin in HeLa cells and HeLa cells expressing GFP-Nup98, GFP-Nup98-HOXA9, GFP-Nup98-JARID1A, respectively. Actin was used as loading control. (PDF) [file pone.0152321.s005.pdf]

**A**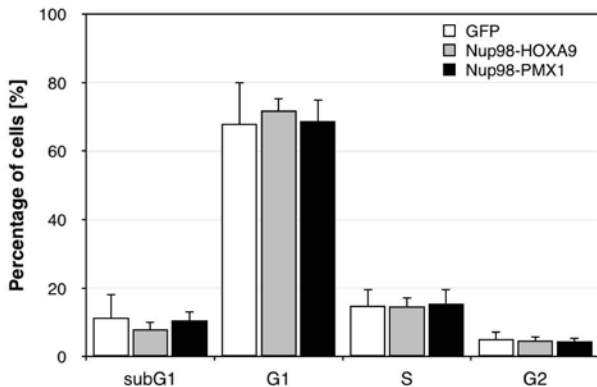**B**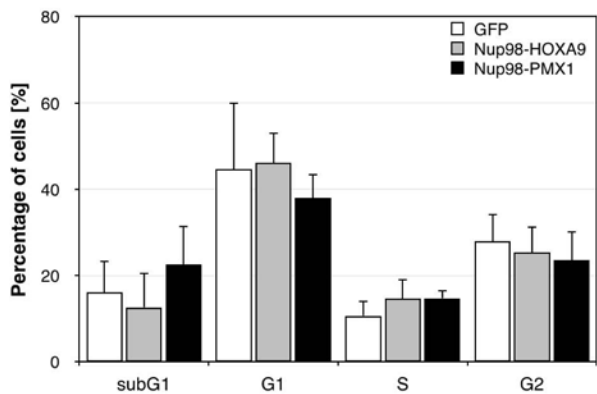

Supplement: S7 Fig — (PDF) [file pone.0152321.s007.pdf]
