# Supplementary material for: Expression of Leukemia-Associated Nup98 Fusion Proteins Generates an Aberrant Nuclear Envelope Phenotype
Source: PLoS One. 2016 Mar 31;11(3):e0152321. doi: 10.1371/journal.pone.0152321 (PMC4816316; doi:10.1371/journal.pone.0152321)
Supplement: S6 Fig — (PDF) [file pone.0152321.s006.pdf]

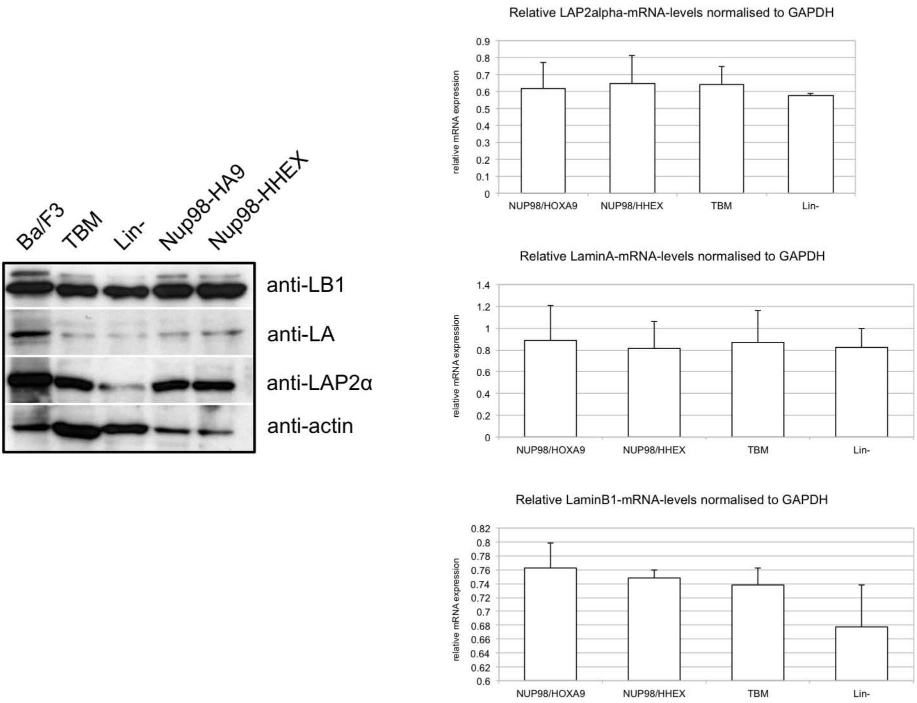
Ba/F3
TBM
Lin-
Nup98-HA9
Nup98-HHEX
anti-LB1
anti-LA
anti-LAP2α
anti-actin
Relative LAP2alpha-mRNA-levels normalised to GAPDH
relative mRNA expression
0.9
0.8
0.7
0.6
0.5
0.4
0.3
0.2
0.1
0
NUP98/HOXA9
NUP98/HHEX
TBM
Lin-
Relative LaminA-mRNA-levels normalised to GAPDH
relative mRNA expression
1.4
1.2
1
0.8
0.6
0.4
0.2
0
NUP98/HOXA9
NUP98/HHEX
TBM
Lin-
Relative LaminB1-mRNA-levels normalised to GAPDH
relative mRNA expression
0.82
0.8
0.78
0.76
0.74
0.72
0.7
0.68
0.66
0.64
0.62
0.6
NUP98/HOXA9
NUP98/HHEX
TBM
Lin-
