# Supplementary material for: Expression of Leukemia-Associated Nup98 Fusion Proteins Generates an Aberrant Nuclear Envelope Phenotype
Source: PLoS One. 2016 Mar 31;11(3):e0152321. doi: 10.1371/journal.pone.0152321 (PMC4816316; doi:10.1371/journal.pone.0152321)
Supplement: S1 Table — (DOCX) [file pone.0152321.s008.docx]

**S1 Table: Plasmids used in this study**

| **Plasmid** | **Construct** | **Source** |
| --- | --- | --- |
| PBF377 | pEGFP-c1-Nup98 | Jankovic et al.; [[32](#_ENREF_32)] |
| PBF378 | pEGFP-c1-Nup98-HOXA9 | Jankovic et al.; [[32](#_ENREF_32)] |
| PBF379 | pEGFP-c1- Nup98-HHEX | Jankovic et al.; [[32](#_ENREF_32)] |
| PBF381 | pEGFP-c1- Nup98-HHEX ∆GLFG | Jankovic et al.; [[32](#_ENREF_32)] |
| PBF382 | pEGFP-c1- Nup98-HHEX ∆HD | Jankovic et al.; [[32](#_ENREF_32)] |
| PBF484 | pEGFP-c1- Nup98-NSD1 | This study |
| PBF528 | pEGFP-c1- Nup98-PMX1 | This study |
| PBF529 | pEGFP-c1-Nup98-NSD3 | This study |
| PBF577 | pEGFP-c1-Nup98-HOXA9 ∆FG | This study |
| PBF590 | pcDNA4-GFP-Nup98-HOXA9 | This study |
| PBF591 | pcDNA4-GFP-Nup98 | This study |
| PBF625 | pEGFP-c1-Nup98-LEDGF | This study |
| PBF636 | pEGFP-c1-Nup98-HOXA9 N51S | This study |
| PBF712 | pEGFP-c1-Nup98-HOXA10 | This study |
| PBF753 | pEGFP-c1- Nup98-PMX1 N51S | This study |
| PBF779 | pEGFP-c1- Nup98-RARG | This study |
| PBF780 | pEGFP-c1- Nup98-JARID1A | This study |
| PBF781 | pEGFP-c1- Nup98-PHF23 | This study |
| PBF783 | pEGFP-c1- Nup98-JARID1A W1625A | This study |
| PBF530 | pEGFP-c1-HHEX | This study |
| PBF531 | pEGFP-c1-HOXA9 | This study |
| PBF538 | pcDNA-HA-AML1-ETO | Addgene plasmid 12508; [[71](#_ENREF_71)] |
|  | pMSCV-Nup98/HOXA9-neo | Jankovic et al.; [[32](#_ENREF_32)] |
|  | pMSCV-Nup98/HHEX-neo | Jankovic et al.; [[32](#_ENREF_32)] |

71. Biggs JR, Peterson LF, Zhang Y, Kraft AS, Zhang DE. AML1/RUNX1 phosphorylation by cyclin-dependent kinases regulates the degradation of AML1/RUNX1 by the anaphase-promoting complex. Mol Cell Biol. 2006;26(20):7420-9. doi: 10.1128/MCB.00597-06. PubMed PMID: 17015473; PubMed Central PMCID: PMC1636878.
