# Supplementary material for: Expression of Leukemia-Associated Nup98 Fusion Proteins Generates an Aberrant Nuclear Envelope Phenotype
Source: PLoS One. 2016 Mar 31;11(3):e0152321. doi: 10.1371/journal.pone.0152321 (PMC4816316; doi:10.1371/journal.pone.0152321)
Supplement: S2 Table — (DOCX) [file pone.0152321.s009.docx]

**S2 Table: qRT-PCR Primer**

| **Target** | **Sense** | **Sequence** |
| --- | --- | --- |
| GAPDH | FWD | TGCACCACCAACTGCTTAG |
|  | REV | GTTCAGCTCAGGGATGACC |
| 28S | FWD | GTTCACCCACTAATAGGGAACGTGA |
|  | REV | GGATTCTGACTTAGAGGCGTTCAGT |
| Cyclin D1 | FWD | CCTGTCCTACTACCGCCTCA |
|  | REV | TCCTCCTCTTCCTCCTCCTC |
| Cyclin E1 | FWD | CAGATTGCAGAGCTGTTGGA |
|  | REV | TCCCCGTCTCCCTTATAACC |
| Bcl-2 | FWD | GAGGATTGTGGCCTTCTTTG |
|  | REV | ACAGTTCCACAAAGGCATCC |
| PCNA | FWD | GGCGTGAACCTCACCAGTAT |
|  | REV | TCTCGGCATATACGTGCAAA |
| TK1 | FWD | GCCAAAGACACTCGCTACAG |
|  | REV | CCCCTCGTCGATGCCTATG |
| RB1 | FWD | TTGGATCACAGCGATACAAACTT |
|  | REV | AGCGCACGCCAATAAAGACAT |
| Lamin A | FWD | CCGAGTCTGAAGAGGTGGTC |
|  | REV | AGGTCACCCTCCTTCTTGGT |
| LAP2α | FWD | GCAGGCAGACATTAGTCAAGC |
|  | REV | CGACCTACAGTGGCATTTCC |
| mouse lamin A | FWD | AGCAAAGTGCGTGAGGAGTT |
|  | REV | GCAGCTTCCTTGGAGTTGAG |
| mouse LAP2α | FWD | CCCGACTTCTCGAGCGAC |
|  | REV | TTTGTGGCTTTCCTGCCGAC |
| mouse GAPDH | FWD | tccactcacggcaaattcaa |
|  | REV | accaggaaatgagcttgacaaa |
| mouse lamin B1 | FWD | AGGCTCTCTACGAGACCGAG |
|  | REV | TCTTGGCATAATTGAGCAGCAG |
|  |  |  |
