# Supplementary material for: Expression of Leukemia-Associated Nup98 Fusion Proteins Generates an Aberrant Nuclear Envelope Phenotype
Source: PLoS One. 2016 Mar 31;11(3):e0152321. doi: 10.1371/journal.pone.0152321 (PMC4816316; doi:10.1371/journal.pone.0152321)
Supplement: S3 Table — AML, acute myeloid leukemia; RAEB, refractory anemia with excess of blast; T-ALL, T-cell acute lymphoblastic leukemia. (DOCX) [file pone.0152321.s010.docx]

**S3 Table: Hematological and cytogenetic features of patient samples**

| **Cases** | **Diagnosis** | **Karyotype** | **Fusion** |
| --- | --- | --- | --- |
| 1 | T-ALL | 47, XX, t(4;11)(q21;p15), +8[20] | NUP98-RAP1GDS1 |
| 2 | RAEB | 46, XY, add(11)(p15)[17]/46, XY [3] | NUP98-NSD1 |
| 3 | AML | 35-45; XY, -1,-9,-10, inv(11)(P15q22),-15, -17,-18,-19,-20,-21[cp11] | NUP98-DDX10 |
|  |  |  |  |

AML, acute myeloid leukemia; RAEB, refractory anemia with excess of blast; T-ALL, T-cell acute lymphoblastic leukemia.
